# An Enhanced Biometric Based Authentication with Key-Agreement Protocol for Multi-Server Architecture Based on Elliptic Curve Cryptography

**DOI:** 10.1371/journal.pone.0154308

**Published:** 2016-05-10

**Authors:** Alavalapati Goutham Reddy, Ashok Kumar Das, Vanga Odelu, Kee-Young Yoo

**Affiliations:** 1 School of Computer Science and Engineering, Kyungpook National University, Daegu, Korea; 2 Center for Security, Theory and Algorithmic Research, International Institute of Information Technology, Hyderabad, India; 3 Department of mathematics, Indian Institute of Technology, Kharagpur, India; Beihang University, CHINA

## Abstract

Biometric based authentication protocols for multi-server architectures have gained momentum in recent times due to advancements in wireless technologies and associated constraints. Lu et al. recently proposed a robust biometric based authentication with key agreement protocol for a multi-server environment using smart cards. They claimed that their protocol is efficient and resistant to prominent security attacks. The careful investigation of this paper proves that Lu et al.’s protocol does not provide user anonymity, perfect forward secrecy and is susceptible to server and user impersonation attacks, man-in-middle attacks and clock synchronization problems. In addition, this paper proposes an enhanced biometric based authentication with key-agreement protocol for multi-server architecture based on elliptic curve cryptography using smartcards. We proved that the proposed protocol achieves mutual authentication using Burrows-Abadi-Needham (BAN) logic. The formal security of the proposed protocol is verified using the AVISPA (Automated Validation of Internet Security Protocols and Applications) tool to show that our protocol can withstand active and passive attacks. The formal and informal security analyses and performance analysis demonstrates that the proposed protocol is robust and efficient compared to Lu et al.’s protocol and existing similar protocols.

## Introduction

The swift expansion of communication technologies and handheld devices have necessitated the authentication of every remote user. Authentication process verifies the legitimacy of each user and offers the access to network resources. Password, smartcard and biometrics based authentication are the few common technologies deployed until today. The first remote user password based authentication method was proposed by Lamport [1] in 1981 for communication over insecure channels. However, password based authentication methods are elusive and prone to guessing attacks. Thus, the password with smartcard based methods have come into sight. Conversely, research has shown that password with smartcard based authentication methods are still prone to numerous attacks when the smartcard is stolen. The ascribed limitations of password and smartcard based authentication methods have imposed to install additional security methods such as biometrics. Biometric keys such as palm print, iris, finger print, face and so on are unique and secure. Biometrics with smartcards or passwords makes the authentication process very robust due to the following features [2] [3]:

- Biometric keys are non-forgeable and non-distributable.
- Biometric keys cannot be lost nor forgotten.
- It is extremely difficult to guess biometric keys unlike passwords.
- Breaking someone’s biometrics is extremely difficult.

Few authentication technologies have used smartcards or biometrics or the both along with passwords [4–23]. Earlier authentication methods were limited to single-server architecture. This architecture is not adequate when the number of users with varied interests and open networks keep increasing. On the other hand, users are required to register at every server in order to avail the services, which is extremely tedious and adds the cost enormously. As a scalable solution, multi-server architecture has been introduced, where the users can register only once at the registration server and avail the services of all associated application servers. Several authors have suggested various authentication protocols for multi-server architecture during the past decade [24–46].

In 2009, Liao et al. [38] proposed a secure dynamic ID based remote user authentication protocol for multi-server environment. In the same year, Hsiang et al. [28] presented that Liao & Wang’s protocol is prone to server and registration center spoofing attacks, insider attacks and masquerade attacks. Furthermore, they proposed an improved dynamic identity based mutual authentication without verification tables. In 2011, Sood et al. [42] proved that Hsiang et al.’s protocol is also vulnerable to impersonation attacks, stolen smart card attacks and replay attacks. In addition, they improved the weaknesses of Hsiang et al.’s protocol and proposed a protocol with different levels of trust between two-servers. In 2012, Li et al. [35] found that Sood et al.’s protocol is susceptible to impersonation attacks, stolen smart card attacks and leak-of-verifier attacks. Then, they proposed an efficient dynamic identity based authentication protocol with smart cards and claimed that it overcomes all aforementioned drawbacks. However, in 2014, Xue et al. [45] proved that Li et al.’s protocol still cannot resist forgery attacks, eavesdropping attacks, denial-of-service attacks and so on. They even put forward a lightweight dynamic pseudonym identity based authentication and key agreement protocol without verification tables for multi-server architecture. In the same year, Chuang et al. [25] proposed an anonymous multi-server authenticated key agreement protocol based on trust computing using smartcards and biometrics. Their protocol is light-weight and provides multi-server authentication with user anonymity. Later on, Mishra et al. [2] in 2014 and Lin et al. [39] in 2015 pointed out several drawbacks of Chuang et al.’s protocol and proposed a secure anonymous three factor authentication protocol. In 2015, Lu et al. [40] proved that Mishra et al.’s protocol was too vulnerable to replay attacks and contains an insecure password changing phase. They proposed a robust biometric based authentication protocol for multi-server architecture.

### Contributions of the paper

Achieving several security properties while maintaining the best performance is essential for any user authentication protocol. Several recent proposed protocols fail to satisfy security and performance properties. One of such protocols is Lu et al.’s robust biometric based authentication protocol for multi-server architecture. This paper’s keen analysis demonstrates the weaknesses of Lu et al.’s protocol such as lack of user anonymity, prone to server and user impersonation attacks, man-in-middle attacks, no perfect forward secrecy and clock synchronization problems. In addition, this paper proposes an enhanced biometric based remote user authentication with key agreement protocol for multi-server architecture without user verification tables. The proposed protocol is perfectly suitable for real time applications as it accomplishes simple elliptic curve cryptography operations, one-way hash functions, concatenation operations and exclusive-OR operations. The proposed protocol is not only light-weight but also achieves all the eminent security properties such as user anonymity, mutual authentication, no verification tables, perfect forward secrecy and resistance to numerous attacks. We proved that the proposed protocol can achieve mutual authentication using BAN logic [47] and the formal security of the proposed protocol is verified using the widely accepted AVISPA tool [48] to ensure the resistance to active and passive attacks. The security and performance analysis sections demonstrates that the proposed protocol is more robust and efficient than Lu et al.’s protocol and other existing protocols.

### Organization of the paper

The remainder of the paper is organized as follows: Section 2 shows the preliminaries used in this paper. Section 3 provides the review of Lu et al.’s protocol. Section 4 crypt analyses Lu et al.’s protocol. Section 5 presents the proposed protocol. Section 6 portrays formal security analysis using BAN logic and informal security analysis of the proposed protocol in detail. In Section 7, the simulation for the formal security verification of the proposed protocol using the AVISPA tool shows that the proposed protocol is secure. Section 8 affords performance analysis and comparison with the related protocols. At last, Section 9 concludes the paper.

## Review of Lu et al.’s Protocol

This section provides an overview of Lu et al.’s [40] biometrics based authentication with key-agreement protocol for multi-server architecture using smartcards. Lu et al.’s protocol comprises three participants, user (*U*_*i*_), authorized server (*S*_*j*_), registration center (*RC*) and four phases, registration phase, login phase, authentication phase, and password change phase. *RC* initializes the system by sharing the chosen secret key *PSK* and random number *x* with *S*_*j*_ via a secure channel. The various notations used in Lu et al.’s protocol are listed in Table 1.

**Table 1:** Notations of Lu et al.’s protocol.

*U*_*i*_	An user
*S*_*j*_	Authorized server
*RC*	Registration center
*ID*_*i*_	Identity of *U*_*i*_
*PW*_*i*_	Password of *U*_*i*_
*BIO*_*i*_	Biometrics of *U*_*i*_
*x*, *y*	Private keys of *RC* and *U*_*i*_
*PSK*	A secure key chosen by *RC* for *S*_*j*_
*h*(.)	A secure one-way hash function
*H*(.)	A bio-hash function
⊕	An exclusive-OR operation
||	The concatenation operation

### Registration phase

**Fig 1:**
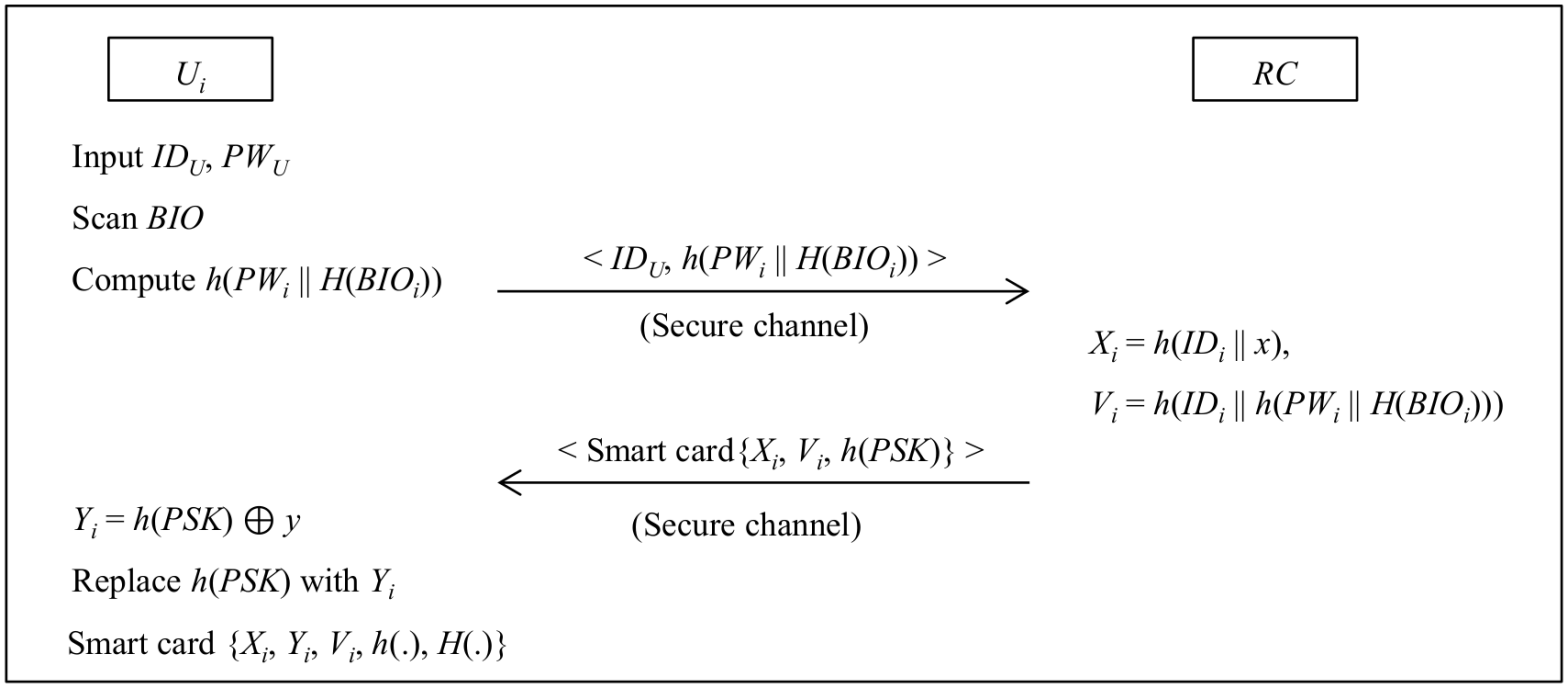
Registration phase of Lu et al.’s protocol.

User (*U*_*i*_) can register at registration center (*RC*) for the first time as shown in Fig 1.

Step 1: *U*_*i*_ chooses an identity *ID*_*i*_, password *PW*_*i*_ and computes *h*(*PW*_*i*_ || *H*(*BIO*_*i*_)). Then sends a request message < *ID*_*i*_, *h*(*PW*_*i*_ || *H*(*BIO*_*i*_)) > to *RC* via a secure channel.
Step 2: *RC* computes *X*_*i*_ = *h*(*ID*_*i*_ || *x*), *V*_*i*_ = *h*(*ID*_*i*_ || *h*(*PW*_*i*_ || *H*(*BIO*_*i*_))). Then *RC* stores the parameters {*X*_*i*_, *V*_*i*_, *h*(*PSK*)} on a smartcard and delivers it to *U*_*i*_ via a secure channel.
Step 3: Upon receiving the smartcard from *RC*, *U*_*i*_ computes *Y*_*i*_ = *h*(*PSK*) ⊕ *y*, and replaces *h*(*PSK*) with *Y*_*i*_. Thus, the smartcard contains {*X*_*i*_, *Y*_*i*_, *V*_*i*_, *h*(.)}.

### Login and authentication phases

**Fig 2:**
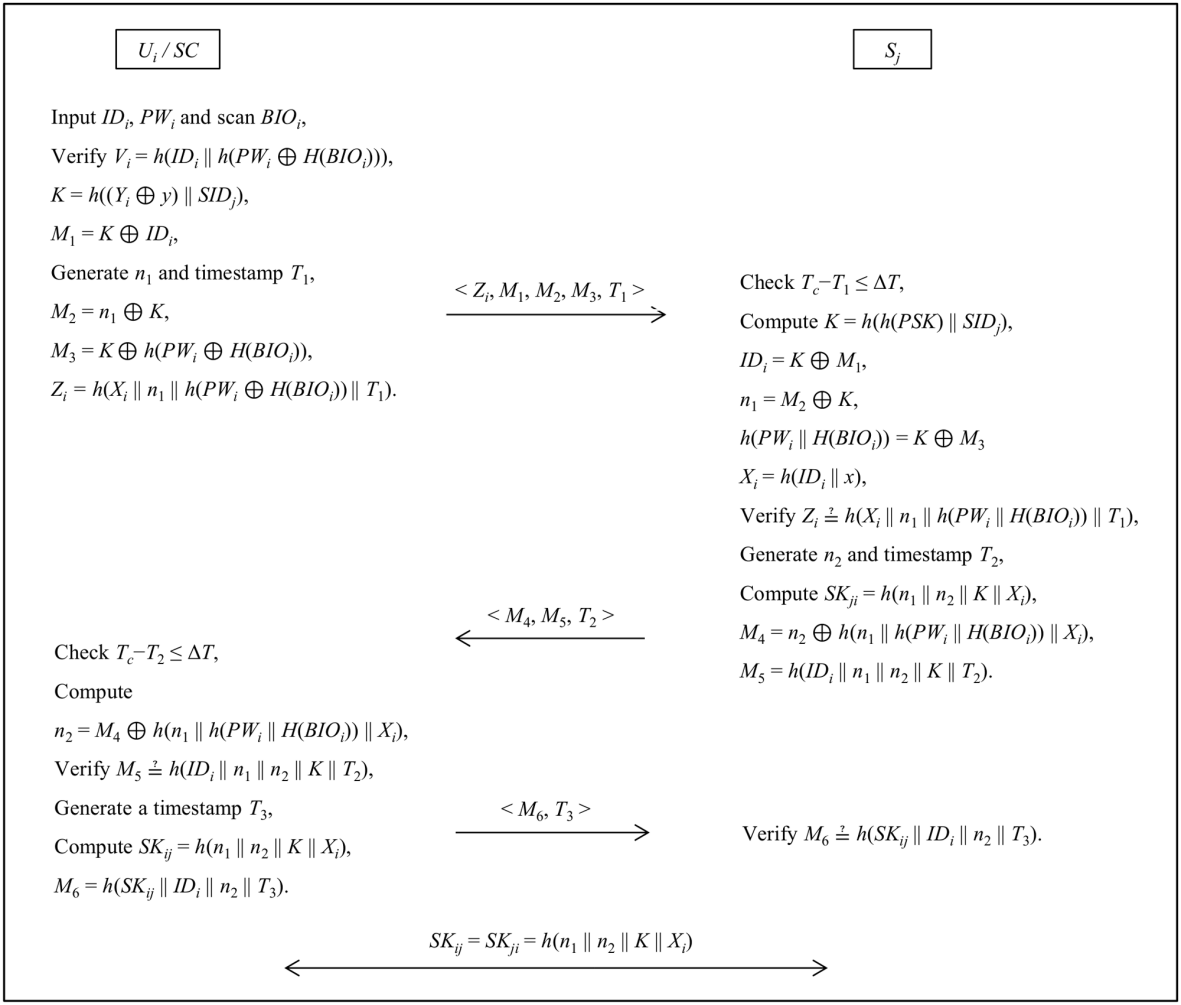
Login and authentication phases of Lu et al.’s protocol.

In this phase, user (*U*_*i*_) and server (*S*_*j*_) authenticates each other, and also establishes a session between them as shown in Fig 2. *U*_*i*_ can launch the login request by inserting smartcard, inputs *ID*_*i*_, *PW*_*i*_ and *BIO*_*i*_.

Step 1: Smartcard computes *h*(*PW*_*i*_ || *H*(*BIO*_*i*_)) and then verifies the condition *V*_*i*_ ≟ *h*(*ID*_*i*_ || *h*(*PW*_*i*_ || *H*(*BIO*_*i*_))). If it generates negative result, the login request can be terminated.
Step 2: Smartcard generates a random number *n*_1_, timestamp *T*_1_ and computes *K* = *h*((*Y*_*i*_ ⊕ *y*) || *SID*_*j*_), *M*_1_ = *K* ⊕ *ID*_*i*_, *M*_2_ = *n*_1_ ⊕ *K*, *M*_3_ = *K* ⊕ *h*(*PW*_*i*_ || *H*(*BIO*_*i*_)), *Z*_*i*_ = *h*(*X*_*i*_ || *n*_1_ || *h*(*PW*_*i*_ || *H*(*BIO*_*i*_)) || *T*_1_), and sends the request message < *Z*_*i*_, *M*_1_, *M*_2_, *M*_3_, *T*_1_ > to *S*_*j*_.
Step 3: *S*_*j*_ checks the freshness of the request message by verifying *T*_*c*_−*T*_1_ ≤ Δ*T*. If it holds, then *S*_*j*_ computes *K* = *h*(*h*(*PSK*) || *SID*_*j*_)) to retrieve *ID*_*i*_ = *K* ⊕ *M*_1_, *n*_1_ = *M*_2_ ⊕ *K*, *h*(*PW*_*i*_ || *H*(*BIO*_*i*_)) = *K* ⊕ *M*_3_. Now, *S*_*j*_ computes *X*_*i*_ = *h*(*ID*_*i*_ || *x*) and verifies *Z*_*i*_ ≟ *h*(*X*_*i*_ || *n*_1_ || *h*(*PW*_*i*_ || *H*(*BIO*_*i*_)) || *T*_1_). If the condition holds, then *S*_*j*_ authenticates *U*_*i*_, otherwise process aborts.
Step 4: *S*_*j*_ further generates a random number *n*_2_, timestamp *T*_2_ and computes *SK*_*ji*_ = *h*(*n*_1_ || *n*_2_ || *K* || *X*_*i*_), *M*_4_ = *n*_2_ ⊕ *h*(*n*_1_ || *h*(*PW*_*i*_ || *H*(*BIO*_*i*_)) || *X*_*i*_), *M*_5_ = *h*(*ID*_*i*_ || *n*_1_ || *n*_2_ || *K* || *T*_2_). *S*_*j*_ sends the response < *M*_4_, *M*_5_, *T*_2_ > to *U*_*i*_.
Step 5: *U*_*i*_ checks the freshness of the message by verifying *T*_*c*_−*T*_2_ ≤ Δ*T*. If it holds, then *U*_*i*_ computes *n*_2_ = *M*_4_ ⊕ *h*(*n*_1_ || *h*(*PW*_*i*_ || *H*(*BIO*_*i*_)) || *X*_*i*_) and checks *M*_5_ ≟ *h*(*ID*_*i*_ || *n*_1_ || *n*_2_ || *K* || *T*_2_). If it generates positive result, then *U*_*i*_ authenticates *S*_*j*_, otherwise process aborts.
Step 6: *U*_*i*_ generates a timestamp *T*_3_ and computes *SK*_*ij*_ = *h*(*n*_1_ || *n*_2_ || *K* || *X*_*i*_), *M*_6_ = *h*(*SK*_*ij*_ || *ID*_*i*_ || *n*_2_ || *T*_3_). Finally, *U*_*i*_ sends < *M*_6_, *T*_3_ > to *S*_*j*_.
Step 7: *S*_*j*_ verifies the freshness of *T*_3_ and *M*_6_ ≟ *h*(*SK*_*ij*_ || *ID*_*i*_ || *n*_2_ || *T*_3_). If it holds, then the mutual authentication with key agreement process between *U*_*i*_ and *S*_*j*_ is completed.

### Password changing phase

A user (*U*_*i*_) can update his/her existing password with new one without the help of registration center (*RC*) as explained below.

Step 1: *U*_*i*_ inserts smartcard, inputs the identity *ID*_*i*_, password *PW*_*i*_, scans the biometrics *BIO*_*i*_ and then verifies the condition *V*_*i*_ ≟ *h*(*ID*_*i*_ || *h*(*PW*_*i*_ || *H*(*BIO*_*i*_))). If it holds, then *U*_*i*_ is allowed to choose a new password *PW*_*i*_^*new*^.
Step 2: Smartcard computes *V*_*i*_^*new*^ ≟ *h*(*ID*_*i*_ || *h*(*PW*_*i*_^*new*^ || *H*(*BIO*_*i*_))) and replaces existing *V*_*i*_ with *V*_*i*_^*new*^.

## Cryptanalysis of Lu et al.’s Protocol

This section cryptanalyses Lu et al.’s [40] protocol and provides a detailed discussion of all security limitations. Lu et al. asserted that their protocol can withstand several renowned attacks while achieving important security features. Conversely, this section proves that their protocol consists of significant drawbacks.

*Limitation* 1: Prone to server impersonation attack

In Lu et al.’s protocol, an adversary *Ӕ* can impersonate as a legitimate server as elucidated here. During server registration phase of Lu et al.’s protocol, registration center *RC* shares the chosen secret key *PSK* and random number *x* with *S*_*j*_ via a secure channel. When an adversary’s server *Ӕ* registers with the *RC*, then after he/she can act as a legitimate server and access all the user’s valuable data due to the possession of common shared attributes *x* and *PSK* in following way:

Step 1: During login and authentication phase, *U*_*i*_ launches the authentication request < *Z*_*i*_, *M*_1_, *M*_2_, *M*_3_, *T*_1_ > by inserting smartcard and inputting *ID*_*i*_, *PW*_*i*_ and *BIO*_*i*_.
Step 2: Upon receiving the request from *U*_*i*_, *Ӕ* computes *K* = *h*(*h*(*PSK*) || *SID*_*j*_)), *ID*_*i*_ = *K* ⊕ *M*_1_, *n*_1_ = *M*_2_ ⊕ *K*, *h*(*PW*_*i*_ || *H*(*BIO*_*i*_)) = *K* ⊕ *M*_3_, *X*_*i*_ = *h*(*ID*_*i*_ || *x*).
Step 3: Now, *Ӕ* generates a random number *n*_2_, timestamp *T*_2_ and computes *SK*_*ji*_ = *h*(*n*_1_ || *n*_2_ || *K* || *X*_*i*_), *M*_4_ = *n*_2_ ⊕ *h*(*n*_1_ || *h*(*PW*_*i*_ || *H*(*BIO*_*i*_)) || *X*_*i*_), *M*_5_ = *h*(*ID*_*i*_ || *n*_1_ || *n*_2_ || *K* || *T*_2_). *Ӕ* sends the response < *M*_4_, *M*_5_, *T*_2_ > to *U*_*i*_.
Step 4: *U*_*i*_ checks the freshness of the message by computing *T*_*c*_−*T*_2_ ≤ Δ*T*. If it holds, then *U*_*i*_ computes *n*_2_ = *M*_4_ ⊕ *h*(*n*_1_ || *h*(*PW*_*i*_ || *H*(*BIO*_*i*_)) || *X*_*i*_) and verifies *M*_5_ ≟ *h*(*ID*_*i*_ || *n*_1_ || *n*_2_ || *K* || *T*_2_). It is obvious that the condition holds, consequently *U*_*i*_ treats *Ӕ* as legitimate *S*_*j*_.
Step 5: *U*_*i*_ generates a timestamp *T*_3_ and computes *SK*_*ij*_ = *h*(*n*_1_ || *n*_2_ || *K* || *X*_*i*_), *M*_6_ = *h*(*SK*_*ij*_ || *ID*_*i*_ || *n*_2_ || *T*_3_). Finally, *U*_*i*_ sends < *M*_6_, *T*_3_ > to *Ӕ*.

Now, *U*_*i*_ may start the communication with *Ӕ* using the computed session key *SK*_*ij*_ = *h*(*n*_1_ || *n*_2_ || *K* || *X*_*i*_) but then is unaware of impersonation attack by *Ӕ*.

*Limitation* 2: Prone to man-in-middle attack

Lu et al.’s protocol is susceptible to man-in-middle attack while disclosing user’s personal valuable data such as *ID*_*i*_ and *h*(*PW*_*i*_ || *H*(*BIO*_*i*_)) as presented here. Assume a legitimate user who contains *h*(*PSK*) becomes an adversary *Ӕ*, then he/she can cause possible damage to the system which is explained in the prone to user impersonation attack subsection.

Step 1: Consider a scenario where *Ӕ* capture the *U*_*i*_’s message < *Z*_*i*_, *M*_1_, *M*_2_, *M*_3_, *T*_1_ > while sending to *S*_*j*_ during authentication phase.
Step 2: *Ӕ* can compute *K* = *h*(*h*(*PSK*) || *SID*_*j*_)) and obtain *ID*_*i*_ = *K* ⊕ *M*_1_, *n*_1_ = *M*_2_ ⊕ *K*, *h*(*PW*_*i*_ || *H*(*BIO*_*i*_)) = *K* ⊕ *M*_3_ by using *h*(*PSK*) and openly available *SID*_*j*_ values.
Step 3: Now *Ӕ* comprises *U*_*i*_’s personal identifiable information such as *ID*_*i*_ and *h*(*PW*_*i*_ || *H*(*BIO*_*i*_)) which are very unique.

*Limitation* 3: Prone to user impersonation attack

In a remote user communication protocol, anyone shall be treated as a legitimate user of the network if he/she has valid authentication credentials or could be able to construct a valid authentication request message. In Lu et al.’s protocol, an adversary *Ӕ* can impersonate a valid user as explained below.

Step 1: As enlightened in prone to server impersonation attack and man-in-middle subsections, *Ӕ* can obtain *U*_*i*_’s personal identifiable information such as *ID*_*i*_ and *h*(*PW*_*i*_ || *H*(*BIO*_*i*_)), and possesses *x* and *PSK* values.
Step 2: Now, *Ӕ* generates a random number *n*_1_, timestamp *T*_1_ and computes *K* = *h*(*h*(*PSK*) || *SID*_*j*_)), *M*_1_ = *K* ⊕ *ID*_*i*_, *M*_2_ = *n*_1_ ⊕ *K*, *M*_3_ = *K* ⊕ *h*(*PW*_*i*_ || *H*(*BIO*_*i*_)), *X*_*i*_ = *h*(*ID*_*i*_ || *x*), *Z*_*i*_ = *h*(*X*_*i*_ || *n*_1_ || *h*(*PW*_*i*_ || *H*(*BIO*_*i*_)) || *T*_1_). *Ӕ* sends the request < *Z*_*i*_, *M*_1_, *M*_2_, *M*_3_, *T*_1_ > to *S*_*j*_.
Step 3: *S*_*j*_ checks the freshness of the message by computing *T*_*c*_−*T*_1_ ≤ Δ*T*. If it holds, then *S*_*j*_ computes *K* = *h*(*h*(*PSK*) || *SID*_*j*_) to retrieve *ID*_*i*_ = *K* ⊕ *M*_1_, *n*_1_ = *M*_2_ ⊕ *K*, *h*(*PW*_*i*_ || *H*(*BIO*_*i*_)) = *K* ⊕ *M*_3_. Then, *S*_*j*_ computes *X*_*i*_ = *h*(*ID*_*i*_ || *x*) and verifies *Z*_*i*_ = *h*(*X*_*i*_ || *n*_1_ || *h*(*PW*_*i*_ || *H*(*BIO*_*i*_)) || *T*_1_). It is obvious that all the conditions generates positive results and *S*_*j*_ treats *Ӕ* as legitimate *U*_*i*_ and proceeds further.
Step 4: *S*_*j*_ generates a random number *n*_2_, timestamp *T*_2_ and computes *SK*_*ji*_ = *h*(*n*_1_ || *n*_2_ || *K* || *X*_*i*_), *M*_4_ = *n*_2_ ⊕ *h*(*n*_1_ || *h*(*PW*_*i*_ || *H*(*BIO*_*i*_)) || *X*_*i*_), *M*_5_ = *h*(*ID*_*i*_ || *n*_1_ || *n*_2_ || *K* || *T*_2_). *S*_*j*_ sends the response < *M*_4_, *M*_5_, *T*_2_ > to *Ӕ*.
Step 5: *Ӕ* computes *n*_2_ = *M*_4_ ⊕ *h*(*n*_1_ || *h*(*PW*_*i*_ || *H*(*BIO*_*i*_)) || *X*_*i*_) and verifies *M*_5_ ≟ *h*(*ID*_*i*_ || *n*_1_ || *n*_2_ || *K* || *T*_2_). Now, *Ӕ* generates a timestamp *T*_3_ and computes *SK*_*ij*_ = *h*(*n*_1_ || *n*_2_ || *K* || *X*_*i*_), *M*_6_ = *h*(*SK*_*ij*_ || *ID*_*i*_ || *n*_2_ || *T*_3_). Finally, *Ӕ* sends < *M*_6_, *T*_3_ > to *S*_*j*_.
Step 6: *S*_*j*_ verifies the freshness of *T*_3_ and *M*_6_ ≟ *h*(*SK*_*ij*_ || *ID*_*i*_ || *n*_2_ || *T*_3_). Since *M*_6_ holds, *S*_*j*_ completes mutual authentication and allows *Ӕ* to access network services.

*Limitation* 4: Lack of user anonymity

Lu et al. claimed that their protocol can achieve one of the important security features called user anonymity. On the contrary, this subsection shows that their protocol cannot hold user anonymity property unlike their claim. During login and authentication phase, *U*_*i*_ transmits the authentication request message < *Z*_*i*_, *M*_1_, *M*_2_, *M*_3_, *T*_1_ > to *S*_*j*_ over public channels. The transmitted parameter *M*_1_ = *K* ⊕ *ID*_*i*_, where *K* = *h*((*Y*_*i*_ ⊕ *y*) || *SID*_*j*_)) in the message < *Z*_*i*_, *M*_1_, *M*_2_, *M*_3_, *T*_1_ > are unique for each user and static during all logins. Hence anyone can track the actions of valid users, if he/she captures *M*_1_ value.

*Limitation* 5: Lack of perfect forward secrecy

Forward secrecy ensures that session key is remaining safe, even if the long term private keys of communicating parties are compromised. In Lu et al. protocol, session key is computed as *SK*_*ij*_ = *h*(*n*_1_ || *n*_2_ || *K* || *X*_*i*_). The involved parameters *K* and *X*_*i*_ are dependent on long term secret keys, and *n*_1_ and *n*_2_ are random numbers. As proved in server impersonation attack, when *S*_*j*_’s long term secret keys *x* and *PSK* are compromised, then *Ӕ* can compute *K* = *h*(*h*(*PSK*) || *SID*_*j*_)), *X*_*i*_ = *h*(*ID*_*i*_ || *x*) and derive session key *SK*_*ij*_ = *h*(*n*_1_ || *n*_2_ || *K* || *X*_*i*_) subsequently. Therefore, Lu et al. protocol does not achieve another vital security feature called perfect forward secrecy.

*Limitation* 6: Prone to clock synchronization problem

Lu et al. uses timestamps to avoid replay attacks on their protocol. The messages < *Z*_*i*_, *M*_1_, *M*_2_, *M*_3_, *T*_1_ > and < *M*_4_, *M*_5_, *T*_2_ > transmitted between *U*_*i*_ and *S*_*j*_ contains timestamps *T*_1_ and *T*_2_. Upon receiving these messages *S*_*j*_ and *U*_*i*_ checks the validity of timestamps by computing *T*_*c*_−*T*_1_ ≤ Δ*T* and *T*_*c*_−*T*_2_ ≤ Δ*T*. If these holds, then only *S*_*j*_ and *U*_*i*_ proceeds further to authenticate each other. In the current world, millions of users contain computing devices due to the greater deployment of networks and technology. It is extremely difficult to synchronize the local system clocks of such a large set of communicating devices. Even a tiny difference in the time could lead to failure of authenticating users. Thus, Lu et al.’s protocol is prone to clock synchronization problem.

## The Proposed Protocol

This section proposes a lightweight biometric based remote mutual authentication with key agreement protocol for multi-server architecture using elliptic curve cryptography. The proposed protocol comprises three participants: user (*U*_*i*_), application server (*AS*), registration server (*RS*) and six phases: registration server initialization phase, application server registration phase, user registration phase, login phase, mutual authentication with key agreement phase, and password and biometrics changing phase. The notations used in the proposed protocol are listed in Table 2.

**Table 2:** Notations of the proposed protocol.

*U*_*i*_	An *i*^*th*^ user
*AS*	Application server
*RS*	Registration server
*ID*_*U*_	Identity of *U*_*i*_
*PW*_*U*_	Password of *U*_*i*_
*b*	A number chosen by *U*_*i*_ for registration
*ID*_*S*_	Identity of *AS*
*USK*	A secure key chosen by *RS* for *U*_*i*_
*PSK*	A secure key chosen by *RS* for *AS*
*x*_*U*_, *x*_*S*_, *N*_1_	Random numbers chosen by *U*_*i*_ and *AS*
*h*(.)	A secure one-way hash function
*H*(.)	A bio-hash function
⊕	An exclusive-OR operation
||	The concatenation operation

### Registration server initialization phase

Registration server (*RS*) generates following parameters in order to initialize the system.

Step 1: *RS* chooses an elliptic curve equation *E* with an order *n*.
Step 2: *RS* selects a base point *P* over *E* and chooses a one-way cryptographic hash function *h*(.).
Step 3: *RS* publishes the information {*E*, *P*, *h*(.)}.

### Application server registration phase

In this phase, application server (*AS*) sends a registration request to the registration server (*RS*) in order to become an authorized server. The application server registration process consists of following steps:

Step 1: *AS* computes public key *R*_*S*_ = *x*_*S*_ *P* sends registration request < *SID*_*S*_, *R*_*S*_ > to the *RS*.
Step 2: *RS* computes *K*_*S*_ = *h*(*SID*_*S*_ || *PSK*), where *PSK* is *RS*’s secret key for application servers and *RS* stores {*SID*_*S*_, *R*_*S*_, *K*_*S*_} in its database table *T*_*US*_.
Step 3: *RS* sends *K*_*S*_ to *AS*, which can be used in further phases of authentication.

### User registration phase

**Fig 3:**
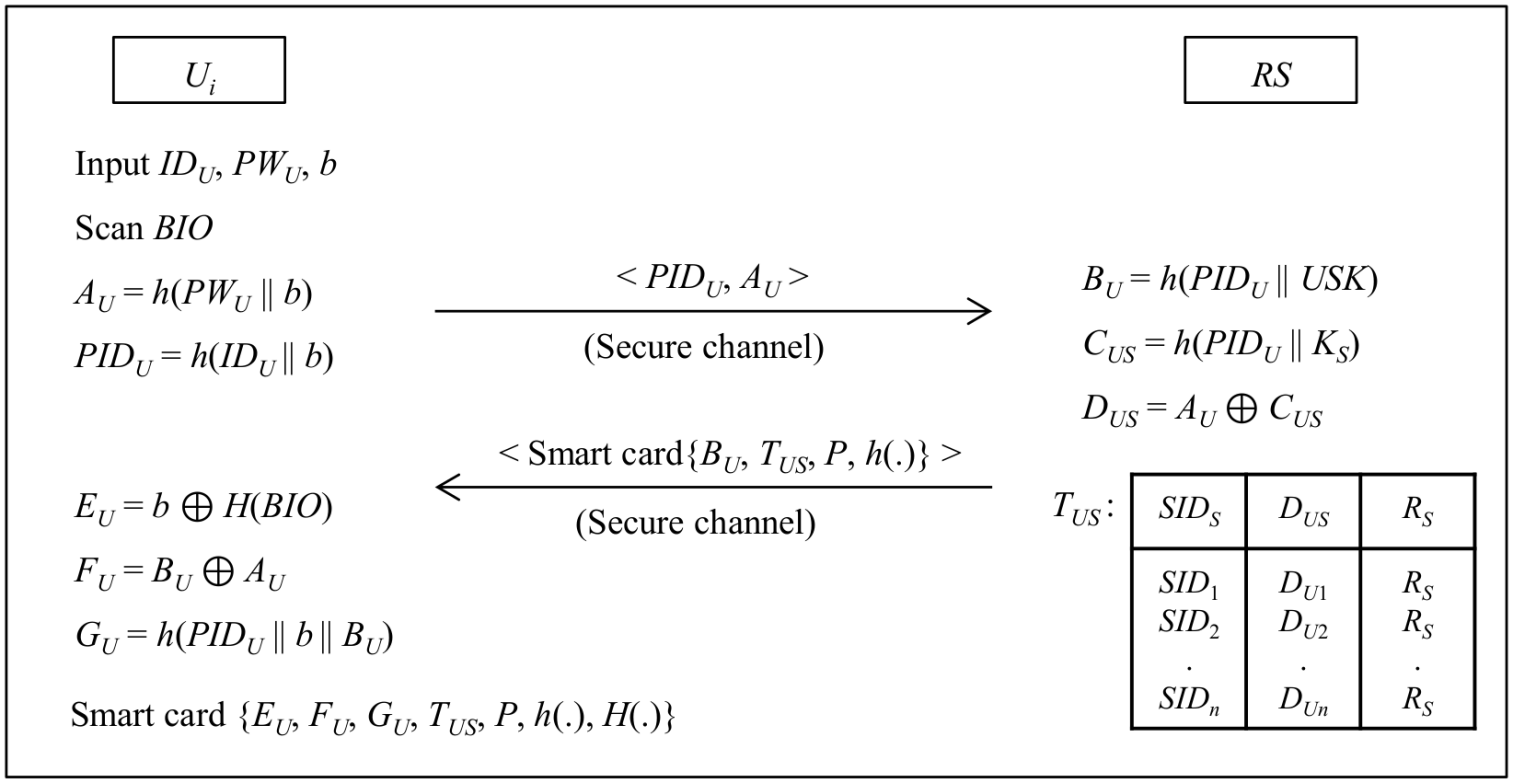
User registration phase.

A new user (*U*_*i*_), who wants to avail the services provided by application servers must register with registration server (*RS*). *U*_*i*_ goes after the following steps to register at *RS* as shown in Fig 3.

Step 1: *U*_*i*_ chooses an identity *ID*_*U*_, password *PW*_*U*_, a number *b* and scans biometrics *BIO* and computes *A*_*U*_ = *h*(*PW*_*U*_ || *b*), *PID*_*U*_ = *h*(*ID*_*U*_ || *b*). *U*_*i*_ sends a request message < *PID*_*U*_, *A*_*U*_ > to *RS* via a secure channel.
Step 2: *RS* computes *B*_*U*_ = *h*(*PID*_*U*_ || *USK*), *C*_*US*_ = *h*(*PID*_*U*_ || *K*_*S*_) and *D*_*US*_ = *A*_*U*_ ⊕ *C*_*US*_, where *USK* is *RS*’s secret key for users.
Step 3: *RS* personalize the parameters {*B*_*U*_, *T*_*US*_, *P*, *h*(.)} on a smartcard and delivers it to *U*_*i*_ via a secure channel.
Step 4: *U*_*i*_ computes *E*_*U*_ = *b* ⊕ *H*(*BIO*), *F*_*U*_ = *B*_*U*_ ⊕ *A*_*U*_, *G*_*U*_ = *h*(*PID*_*U*_ || *b* || *B*_*U*_) and stores *E*_*U*_, *G*_*U*_, *F*_*U*_ on the received smart card after deleting *B*_*U*_ from smartcard. Thus the smartcard finally contains the parameters {*E*_*U*_, *G*_*U*_, *F*_*U*_, *T*_*US*_, *P*, *h*(.), *H*(.)}.

### Login phase

When a user (*U*_*i*_) wants to access the services of application server (*AS*), he/she launches the login request by inserting smartcard (*SC*), and inputting *ID*_*U*_, *PW*_*U*_ and *BIO*.

Step 1: *SC* →*AS*: < *AID*_*U*_, *M*_1_, *R*_*U*_ >

*SC* computes *b* = *E*_*U*_ ⊕ *H*(*BIO*), *A*_*U*_ = *h*(*PW*_*U*_ || *b*), *PID*_*U*_ = *h*(*ID*_*U*_ || *b*), *B*_*U*_ = *F*_*U*_ ⊕ *A*_*U*_ and then verifies the condition *G*_*U*_ ≟ *h*(*PID*_*U*_ || *b* || *B*_*U*_). If it generates negative result, the login request can be terminated. Otherwise, *SC* retrieves corresponding application server’s *D*_*US*_ and *R*_*S*_ values from the table *T*_*US*_. Then, *SC* generates a random number *x*_*U*_ and calculates *R*_*U*_ = *x*_*U*_ *P*, *R*_*U*_′ = *x*_*U*_ *R*_*S*_, *C*_*US*_ = *A*_*U*_ ⊕ *D*_*US*_, *AID*_*U*_ = *PID*_*U*_ ⊕ *R*_*U*_′, *M*_1_ = *h*(*PID*_*U*_ || *C*_*US*_ || *R*_*U*_ || *R*_*U*_′) and sends the login request message < *AID*_*U*_, *M*_1_, *R*_*U*_ > to *AS*.

### Mutual authentication with key-agreement phase

**Fig 4:**
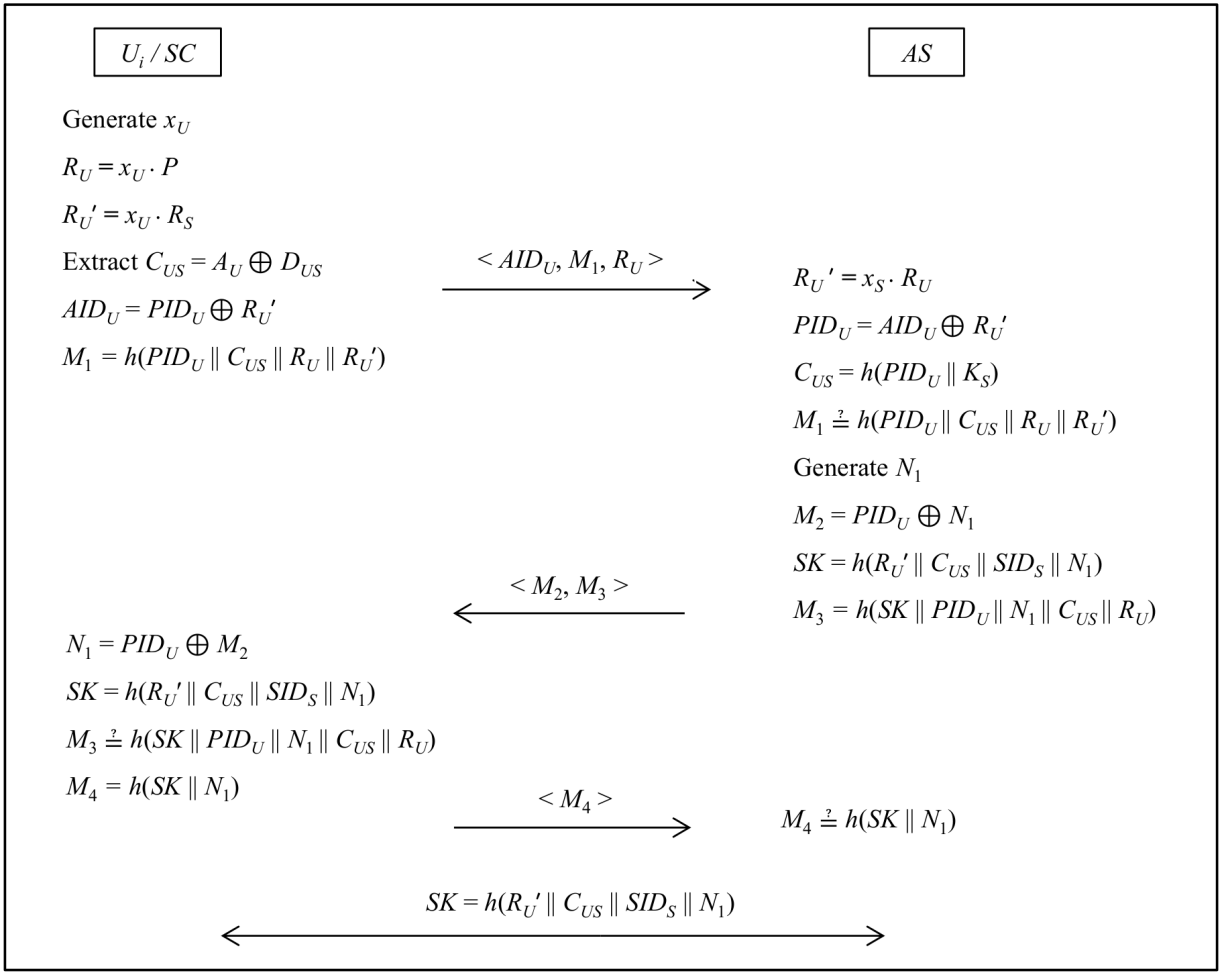
Mutual authentication with key-agreement phase.

In this phase, *U*_*i*_ and *AS* authenticates each other and computes a session key for further secure communication over public channels. The entire mutual authentication with key agreement phase is illustrated in Fig 4.

Step 1: *AS* computes *R*_*U*_′ = *x*_*S*_ *R*_*U*_, *PID*_*U*_ = *AID*_*U*_ ⊕ *R*_*U*_′, *C*_*US*_ = *h*(*PID*_*U*_ || *K*_*S*_) and verifies the condition *M*_1_ ≟ *h*(*PID*_*U*_ || *C*_*US*_ || *R*_*U*_ || *R*_*U*_′). If the condition holds, *AS* authenticates *U*_*i*_, otherwise the process can be terminated.
Step 2: *AS* → *SC*: < *M*_2_, *M*_3_ >
  *AS* further generates a random number *N*_1_ and computes *SK* = *h*(*R*_*U*_′ || *C*_*US*_ || *SID*_*S*_ || *N*_1_), *M*_2_ = *PID*_*U*_ ⊕ *N*_1_, *M*_3_ *= h*(*SK* || *PID*_*U*_ || *N*_1_ || *C*_*US*_ || *R*_*U*_). *AS* sends < *M*_2_, *M*_3_ > to *SC*.
Step 3: *SC* → *AS*: < *M*_4_ >
  *SC* computes *N*_1_ = *PID*_*U*_ ⊕ *M*_2_, *SK* = *h*(*R*_*U*_′ || *C*_*US*_ || *SID*_*S*_ || *N*_1_) and verifies the condition *M*_3_ ≟ *h*(*SK* || *PID*_*U*_ || *N*_1_ || *C*_*US*_ || *R*_*U*_). If the condition holds, *U*_*i*_ authenticates *AS*, otherwise the process can be terminated. Then, *SC* computes *M*_4_ *= h*(*SK* || *N*_1_) and sends it to *AS*.
Step 4: *AS* verifies *M*_4_ ≟ *h*(*SK* || *N*_1_) and reconfirms the authenticity of *U*_*i*_. Now, *U*_*i*_ and *AS* can start communication with the computed session key *SK*.

### Password and biometrics changing phase

This procedure invokes when a user (*U*_*i*_) wish to update his/her existing password with new one. In this procedure, *U*_*i*_ can change his/her password without the involvement of registration server (*RS*) as follows:

Step 1: *U*_*i*_ inserts smartcard *SC* and inputs *ID*_*U*_, *PW*_*U*_ and *BIO*.
Step 2: *SC* computes *b* = *E*_*U*_ ⊕ *H*(*BIO*), *A*_*U*_ = *h*(*PW*_*U*_ || *b*), *PID*_*U*_ = *h*(*ID*_*U*_ || *b*), *B*_*U*_ = *F*_*U*_ ⊕ *A*_*U*_ and then verifies the condition *G*_*U*_ ≟ *h*(*PID*_*U*_ || *b* || *B*_*U*_). If the condition holds, *U*_*i*_ derives *C*_*US*_ = *A*_*U*_ ⊕ *D*_*US*_ for all the servers in the table *T*_*US*_, otherwise request can be dropped.
Step 3: *U*_*i*_ chooses a new password *PW*_*U*_^#^ and *BIO*^#^ and then computes *A*_*U*_^#^ = *h*(*PW*_*U*_^#^ || *b*), *F*_*U*_^#^ = *B*_*U*_ ⊕ *A*_*U*_^#^, *D*_*US*_^#^ = *A*_*U*_^#^ ⊕ *C*_*US*_ and *E*_*U*_^#^ = *b* ⊕ *H*(*BIO*^#^). *U*_*i*_ updates the table *T*_*US*_^#^ and the parameters *F*_*U*_^#^, *E*_*U*_^#^ on the smartcard. Thus the smartcard finally contains the parameters {*E*_*U*_^#^, *F*_*U*_^#^, *G*_*U*_, *T*_*US*_^#^, *P*, *h*(.), *H*(.)}.

## Security Analysis

This section exhibits the security analysis of proposed authentication protocol for multi-server architecture by describing each security feature. This analysis checks various security aspects and ensures that the proposed protocol is resistant to different attacks and certain flaws are not exhibited.

### Formal security analysis using BAN logic

Formal security analysis of the proposed protocol is verified with the help of Burrows-Abadi-Needham (BAN) logic [46]. This section proves that the proposed protocol provides secure mutual authentication between a user *U*_*i*_ and an application server *AS*.

The following notations are used in formal security analysis using the BAN logic:

- *Q* |≡ *X*: Principal *Q* believes the statement *X*.
- #(*X*): Formula *X* is fresh.
- *Q* | ⇒ *X*: Principal *Q* has jurisdiction over the statement *X*.
- *Q* ⊲ *X*: Principal *Q* sees the statement *X*.
- *Q* | ∼ *X*: Principal *Q* once said the statement *X*.
- (*X*, *Y*): Formula *X* or *Y* is one part of the formula (*X*, *Y*).
- ⟨*X*⟩_*Y*_: Formula *X* combined with the formula *Y*.
- $Q\;\overset{K}{↔}\;R$: Principal *Q* and *R* may use the *shared key K* to communicate among each other. The key *K* is good, in that it will never be discovered by any principal except *Q* and *R*.
- $Q\;\overset{X}{\Leftrightarrow }\;R$: Formula *X* is secret known only to *Q* and *R*, and possibly to principals trusted by them.

In addition, the following four BAN logic rules are used to prove that the proposed protocol provides a secure mutual authentication between *U*_*i*_ and *AS*:

- Rule 1. Message-meaning rule: $\frac{R\;|\equiv \;R\overset{Y}{↔}S,\;R\;⊲\;{\langle X\rangle }_{Y}}{R\;\left|\equiv \;S\right|\sim \;X}$ and $\frac{P\;|\equiv \;P\overset{Y}{↔}Q,\;P\;⊲\;{\langle X\rangle }_{Y}}{P\;\left|\equiv \;Q\right|\sim \;X}$
- Rule 2. Nonce-verification rule: $\frac{R\left|\equiv \;\#\left(X\right),\;R\right|\equiv S\;|\sim \;X}{R\;\left|\equiv \;S\right|\equiv \;X}$
- Rule 3. Jurisdiction rule: $\frac{R\;\left|\equiv \;S\right|\Rightarrow X,\;R\;\left|\equiv S\right|\equiv \;X}{R\;|\equiv \;\;X}$
- Rule 4. Freshness-conjuncatenation rule: $\frac{R\;|\equiv \;\#\left(X\right)}{R\;|\equiv \;\#\left(X,Y\right)}$

In order to show that the proposed protocol provides secure mutual authentication between a node *R* in the cluster *C*_*i*_ and *TM*, we need to achieve the following four test goals:

- Goal 1: $U_{i}|\equiv U_{i}\overset{SK}{↔}AS$
- Goal 2: $U_{i}|\equiv \;AS|\equiv U_{i}\overset{SK}{↔}AS$
- Goal 3: $AS|\equiv U_{i}\overset{SK}{↔}AS$
- Goal 4: $AS|\equiv \;U_{i}|\equiv U_{i}\overset{SK}{↔}AS$

**Generic form:** The generic forms of the transmitted messages between the user *U*_*i*_ and the application server *AS* in the proposed protocol are given below:

- M1. *U*_*i*_ → *AS*: *${\langle PID_{U},\;R_{U},R_{U}\text{'}\rangle }_{C_{US}}$*
- M2. *AS* → *U*_*i*_: *${\langle SK,\;PID_{U},\;N_{1},R_{U}\;\rangle }_{C_{US}}$*
- M3. *U*_*i*_ → *AS*: *${\langle R_{U}\text{'},SID_{S},\;N_{1}\;\rangle }_{C_{US}}$*

Note that the message M3, *U*_*i*_ → *AS*: *h*(*h*(*R*_*U*_’, *C*_*US*_, *SID*_*S*_, *N*_1_), *N*_1_) authenticates the parameters *R*_*U*_’, *SID*_*S*_, *N*_1_ under the shared secret *C*_*US*_ between *U*_*i*_ and *AS*. Thus, for simplicity we assume that the message M3 as *U*_*i*_ → *AS*: ${\langle R_{U}\text{'},SID_{S},\;N_{1}\rangle }_{C_{US}}$ in the generic form.

**Idealized form:** The arrangement of the transmitted messages between *U*_*i*_ and *AS* in the proposed protocol to the idealized forms are as follows:

- M1. *U*_*i*_ → *AS*: *${\langle PID_{U},\;R_{U},U_{i}\;\overset{R_{U}\text{'}}{↔}\;AS\rangle }_{U_{i}\overset{C_{US}}{\Leftrightarrow }AS}$*
- M2. *AS* → *U*_*i*_: *${\langle U_{i}\;\overset{SK}{↔}\;AS,\;PID_{U},\;N_{1},R_{U}\rangle }_{U_{i}\overset{C_{US}}{\Leftrightarrow }AS}$*
- M3. *U*_*i*_ → *AS*: *${\langle U_{i}\;\overset{R_{U}\text{'}}{↔}\;AS\;,SID_{S},\;N_{1}\rangle }_{U_{i}\overset{C_{US}}{\Leftrightarrow }AS}$*

**Hypotheses:** The following are the initial assumptions of the proposed protocol:

- H1: *U*_*i*_ |≡ #(*R*_*U*_),
- H2: *AS* |≡ #(*N*_1_),
- H3: $U_{i}|\equiv U_{i}\overset{C_{US}}{↔}AS$,
- H4: $AS|\equiv U_{i}\overset{C_{US}}{↔}AS$,
- H5: $AS|\equiv \;U_{i}|\Rightarrow U_{i}\overset{R_{U}\text{'}}{↔}AS$,
- H6: $U_{i}|\equiv \;AS|\Rightarrow U_{i}\overset{SK}{↔}AS$,
- H7: $U_{i}|\equiv U_{i}\overset{R_{U}\text{'}}{↔}AS$.

In the following, we prove the above test goals in order to show the secure authentication using the BAN logic rules and the assumptions.

- From the message M1, we have, S1: $AS⊲\;{\langle PID_{U},\;R_{U},U_{i}\;\overset{R_{U}\text{'}}{↔}\;AS\;\rangle }_{U_{i}\overset{C_{US}}{\Leftrightarrow }AS}$
- From S1, H4, and Rule 1, we get, S2: $AS\left|\equiv \;U_{i}\right|\;∼\;\langle PID_{U},\;R_{U},U_{i}\;\overset{R_{U}\text{'}}{↔}\;AS\;\rangle$
- From the message M2, we have, S3: $U_{i}⊲\;U_{i}{\langle \;\overset{SK}{↔}\;AS,\;PID_{U},\;N_{1},R_{U}\rangle }_{U_{i}\overset{C_{US}}{\Leftrightarrow }AS}$
- From S3, H3, and Rule 1, we obtain, S4: $U_{i}\left|\equiv AS\right|\;∼\langle \;U_{i}\;\overset{SK}{↔}\;AS,\;PID_{U},\;N_{1},R_{U}\rangle$
- From S4, H1, Rule 2, and Rule 4, we get, S5: $U_{i}\left|\equiv AS\right|\;\equiv U_{i}\;\overset{SK}{↔}\;AS$ (Goal 2)
- From S5 and Jurisdiction rule Rule 3, we obtain, S6: $U_{i}|\equiv U_{i}\;\overset{SK}{↔}\;AS$ (Goal 1)
- From the message M3, we have, S7: $AS\;⊲{\langle U_{i}\;\overset{R_{U}\text{'}}{↔}\;AS\;,SID_{S},\;N_{1}\rangle }_{U_{i}\overset{C_{US}}{\Leftrightarrow }AS}$
- From the message S7, H4, and Rule 1, we have, S8: $AS\;\left|\equiv \;U_{i}\right|\;∼\langle U_{i}\;\overset{R_{U}\text{'}}{↔}\;AS\;,SID_{S},\;N_{1}\rangle$
- From S8, H2, Rule 2, and Rule 4, we get, S9: $AS\;\left|\equiv \;U_{i}\right|\;\equiv U_{i}\;\overset{R_{U}\text{'}}{↔}\;AS$
- Since SK is computed as *SK* = *h*(*h*(*R*_*U*_’, *C*_*US*_, *SID*_*S*_, *N*_1_), *N*_1_), from S9 and H4, we get the required goal Goal 3, as S10: $AS\;\left|\equiv \;U_{i}\right|\;\equiv U_{i}\;\overset{SK}{↔}\;AS$ (Goal 4)
- Finally, from S10 and Jurisdiction rule Rule 3, we obtain, S11: $AS\;|\equiv \;U_{i}\;\overset{SK}{↔}\;AS$ (Goal 3)

### Informal security analysis

#### Proposition 1

The proposed protocol achieves user anonymity and untraceability.

#### Proof

The proposed protocol does not reveal the real identities of users throughout all the phases of communication. In the user registration phase *U*_*i*_ submits pseudonym identity *PID*_*U*_ = *h*(*ID*_*U*_ || *b*) and the real identity is guarded with a one-way hash function. During login phase, the pseudonym identity *PID*_*U*_ is converted as anonymous in the form of *AID*_*U*_ = *PID*_*U*_ ⊕ *R*_*U*_′. The identity is dynamic for every login, due to its association with a randomly chosen number *x*_*U*_, where *R*_*U*_ = *x*_*U*_ *P* and *R*_*U*_′ = *x*_*U*_ *R*_*S*_. An adversary cannot retrieve the user’s pseudonym identity *PID*_*U*_ without having the knowledge of the user’s password *PW*_*U*_ and *b*. Moreover, it is believed to be impossible to compute *R*_*U*_′ from *R*_*U*_ and *R*_*S*_ due to the fact of ECDLP. The proposed protocol provides another important feature called untraceability. An adversary may try to trace the actions of users by observing the transmitting parameters. In the login phase, *U*_*i*_ sends the message < *AID*_*U*_, *M*_1_, *R*_*U*_ > to *AS*. All the parameters are dynamic and does not disclose any information about *U*_*i*_. Thus, the proposed protocol achieves user anonymity with untraceability.

#### Proposition 2

The proposed protocol is secure against replay attacks and clock synchronization problem.

#### Proof

The proposed protocol adopts the method discussed in the recent protocols [2] [3] [24] [25] [36], known as deployment of random numbers to endure replay attack. During mutual authentication and key-agreement phase, *AS* and *U* obtains {*PID*_*U*_, *R*_*U*_} and {*N*_1_} and stores the values in its database tables, respectively. Consider a scenario where an adversary acquire {*AID*_*U*_, *M*_1_, *R*_*U*_} or {*M*_2_, *M*_3_} or {*M*_4_} and replay the same message. However, adversary definitely cannot construct a valid session due to the reason explained here. All the captured parameters are randomized by incorporating a random number *x*_*U*_ in the form of *R*_*U*_ = *x*_*U*_ *P* and *R*_*U*_′ = *x*_*U*_ *R*_*S*_ in *AID*_*U*_ = *PID*_*U*_ ⊕ *R*_*U*_′, *M*_1_ = *h*(*PID*_*U*_ || *C*_*US*_ || *R*_*U*_ || *R*_*U*_′). The random number *x*_*U*_ always keeps the transmitting parameters as dynamic for every session. If an adversary sends < *AID*_*U*_, *M*_1_, *R*_*U*_ > to *AS*, it identifies *R*_*U*_ as previous transmitted message and drops the requested session. In the same way, *N*_1_ helps in identifying the replay attacks of {*M*_2_, *M*_3_} and {*M*_4_}.

In a cryptographic authentication protocol environment, timestamps are used to protect the messages from replay attacks. Basically, timestamps will be generated from the internal clocks of computing systems and may differ from system to system known as, clock synchronization problem. Hence, in the current large network field, time stamps are not the definite solutions due to clock synchronization problems. As an alternative possible solution, the proposed protocol deploys random numbers *x*_*U*_ and *N*_1_.

#### Proposition 3

The proposed protocol is secure against stolen smart card attack.

#### Proof

Reading a smartcard stored values is possible by means of power analysis and various other ways [49] [50] [51]. Assume a valid user’s smartcard is stolen by an adversary and stored parameters {*E*_*U*_, *G*_*U*_, *F*_*U*_, *T*_*US*_, *P*, *h*(.)} on it are extracted. Now, the adversary may try to derive authentication credentials from the extracted parameters. However, adversary undeniably cannot obtain any valuable information from these values, since all the important parameters such as *E*_*U*_ = *b* ⊕ *H*(*BIO*), *F*_*U*_ = *B*_*U*_ ⊕ *A*_*U*_, *G*_*U*_ = *h*(*PID*_*U*_ || *b* || *B*_*U*_) are safeguarded with a one-way hash function, where *PID*_*U*_ = *h*(*ID*_*U*_ || *b*), *A*_*U*_ = *h*(*PW*_*U*_ || *b*) and *B*_*U*_ = *h*(*PID*_*U*_ || *USK*). Note that the identity of user *ID*_*U*_ is not stored on the smartcard. The adversary cannot obtain any login information using the smartcard stored parameters *E*_*U*_, *G*_*U*_, *F*_*U*_. At the same time guessing the real identity *ID*_*U*_ and password *PW*_*U*_ is impractical. Aforementioned constraints proves that the proposed protocol is secure from smartcard stolen attack.

#### Proposition 4

The proposed protocol is secure against user impersonation attack.

#### Proof

Assume a situation where an adversary possesses a valid smartcard and wants to gain network access by perpetrating user impersonation attack. If an adversary wants to impersonate a legitimate user *U*_*i*_, he/she requires to build a login request message < *AID*_*U*_, *M*_1_, *R*_*U*_ >, where *R*_*U*_ = *x*_*U*_ *P*, *AID*_*U*_ = *PID*_*U*_ ⊕ *R*_*U*_′, *M*_1_ = *h*(*PID*_*U*_ || *C*_*US*_ || *R*_*U*_ || *R*_*U*_′). Conversely, the adversary can barely compute two parameters *R*_*U*_^#^ = *x*_*U*_^#^ *P* and *R*_*U*_^#^′ = *x*_*U*_^#^ *R*_*S*_ by choosing his/her own random number *x*_*U*_^*#*^. In order to compute rest of the two parameters, adversary requires user’s identity *ID*_*U*_ and password *PW*_*U*_, which are unobtainable.

On the other hand, the adversary should undergo login phase before making authentication request. During login phase, *SC* computes *b* = *E*_*U*_ ⊕ *H*(*BIO*), *A*_*U*_ = *h*(*PW*_*U*_ || *b*), *PID*_*U*_ = *h*(*ID*_*U*_ || *b*), *B*_*U*_ = *F*_*U*_ ⊕ *A*_*U*_ and then verifies the condition *G*_*U*_ ≟ *h*(*PID*_*U*_ || *b* || *B*_*U*_). Unless the adversary enters the correct credentials, he/she cannot be allowed to further phases. Therefore, the adversary certainly requires legitimate identity *ID*_*U*_ and password *PW*_*U*_ for any furthermore computations. However, the probability of yielding correct *ID*_*U*_ and *PW*_*U*_ is negligible. The adversary may also try to extract *PID*_*U*_ from *AID*_*U*_ = *PID*_*U*_ ⊕ *R*_*U*_′, by guessing *x*_*U*_ from *R*_*U*_ = *x*_*U*_ *P* and *R*_*U*_′ = *x*_*U*_ *R*_*S*_. It is even more difficult to perform the above operation due to the fact of Elliptic Curve Diffie-Hellman Problem (ECDHP).

#### Proposition 5

The proposed protocol is secure against application server impersonation attack.

#### Proof

Usually, during authentication phase, *AS* computes *M*_2_ = *PID*_*U*_ ⊕ *N*_1_, *SK* = *h*(*R*_*U*_′ || *C*_*US*_ || *SID*_*S*_ || *N*_1_), *M*_3_ *= h*(*SK* || *PID*_*U*_ || *N*_1_ || *C*_*US*_ || *R*_*U*_) with the generated random number *N*_1_ and sends < *M*_2_, *M*_3_ > to *U*_*i*_. Consider a scenario where an adversary’s server acts as a legitimate one and proceeds with the authentication and key agreement procedures. In order to compute session key *SK*, adversary must have *R*_*U*_′, *C*_*US*_ and *SID*_*J*_. Assume that adversary still proceeds with the computations *R*_*U*_^*#*^′ = *x*_*S*_^*#*^ *R*_*U*_, *M*_2_^*#*^ = *PID*_*U*_^*#*^ ⊕ *N*_1_^*#*^, *SK*^*#*^ = *h*(*R*_*U*_^*#*^′ || *C*_*US*_^*#*^ || *SID*_*S*_ || *N*_1_^*#*^), *M*_3_^*#*^ *= h*(*SK*^*#*^ || *PID*_*U*_^*#*^ || *N*_1_^*#*^ || *C*_*US*_^*#*^ || *R*_*U*_) with the generated random numbers *N*_1_^*#*^ and *x*_*S*_^*#*^. Note that *SID*_*S*_ can be obtained from the table *T*_*US*_ in the smartcard. Upon receiving the response < *M*_2_^*#*^, *M*_3_^*#*^ >, *U*_*i*_ computes *N*_1_^*#*^ = *PID*_*U*_ ⊕ *M*_2_^*#*^, *SK* = *h*(*R*_*U*_′ || *C*_*US*_ || *SID*_*S*_ || *N*_1_^*#*^) and *M*_3_^*#*^ *= h*(*SK* || *PID*_*U*_ || *N*_1_^*#*^ || *C*_*US*_ || *R*_*U*_) and tallies the received *M*_3_^*#*^ with the computed *M*_3_. Here, *U*_*i*_ identifies it as a fake response from the malicious server due to *M*_3_ ≠ *M*_3_^#^ and terminates the session immediately. Thus, the proposed protocol can withstand application server impersonation attacks.

#### Proposition 6

The proposed protocol is secure against man-in-middle attack.

#### Proof

In the proposed protocol scenario, adversary has the possibility of attacking either request message or response messages as elucidated here. Authentication request message < *AID*_*U*_, *M*_1_, *R*_*U*_ > initiates from *SC* to *AS*. As explained in proposition 4, adversary can modify only one parameter *R*_*U*_^#^ = *x*_*U*_^#^ *P* with the chosen random number *x*_*U*_^*#*^. For instance the adversary sends the modified parameter in the message as < *AID*_*U*_, *M*_1_, *R*_*U*_^#^ >. Upon receiving it, *AS* computes *R*_*U*_^#^′ = *x*_*S*_ *R*_*U*_^#^, *PID*_*U*_^#^ = *AID*_*U*_ ⊕ *R*_*U*_^#^′, *C*_*US*_^#^ = *h*(*PID*_*U*_^#^ || *K*_*S*_) and *M*_1_^#^ = *h*(*PID*_*U*_^#^ || *C*_*US*_^#^ || *R*_*U*_^#^ || *R*_*U*_^#^′). Finally, *AS* compares the received *M*_1_ with the computed *M*_1_^#^ then apparently *M*_1_ ≠ *M*_1_^#^. Accordingly, *AS* identifies it as a malicious attack and acknowledges the user *U*_*i*_. In case, the adversary wants to accomplish active attacks on response messages either < *M*_2_, *M*_3_ > or < *M*_4_ >, then session key *SK* = *h*(*R*_*U*_′ || *C*_*US*_ || *SID*_*S*_ || *N*_1_) value and other parameters are essential and unobtainable. Thus the proposed protocol can withstand a man-in-middle attack.

#### Proposition 7

The proposed protocol is secure against password guessing attack.

#### Proof a

[*Offline password guessing attack*]: An adversary may attempt to guess the password *PW*_*U*_ from the extracted smart card stored parameters {*E*_*U*_, *G*_*U*_, *F*_*U*_, *T*_*US*_, *P*, *h*(.)}. The stored parameter *F*_*U*_ = *B*_*U*_ ⊕ *A*_*U*_ contains the password *PW*_*U*_ in the form *A*_*U*_ = *h*(*PW*_*U*_ || *b*). An adversary can try to check the condition *F*_*U*_ ≟ *B*_*U*_ ⊕ *A*_*U*_ while constantly guessing *PW*_*U*_. In order to execute this, adversary needs *ID*_*U*_ and *b* values as well. However, *ID*_*U*_ value is nowhere stored and *b* value is protected with biometrics *H*(*BIO*), which can neither be forged nor copied. The adversary may even attempt to perform the same on *AID*_*U*_ = *PID*_*U*_ ⊕ *R*_*U*_′ value intercepted from previous login message < *AID*_*U*_, *M*_1_, *R*_*U*_ >. To perform this, the adversary requires *R*_*U*_′ = *x*_*U*_ *R*_*S*_. As a result, the adversary would fail to guess the correct password *PW*_*U*_. Therefore, the proposed protocol is secure against offline password guessing attack.

#### Proof b

[*Online password guessing attack*]: If an adversary possesses the valid smartcard, he/ she may keep trying to login while guessing the password *PW*_*U*_. Unless the adversary passes valid biometrics *BIO*; *b* value cannot be retrieved from *E*_*U*_. Additionally, the login verification condition *G*_*U*_ ≟ *h*(*PID*_*U*_ || *b* || *B*_*U*_) checks the correctness of all input credentials. If the adversary enters the wrong password for certain number of times, the system may abort and would not allow entering credentials for some time. In addition, it is almost impractical to guess all the required values within polynomial time.

#### Proposition 8

The proposed protocol is secure against privileged insider attack and does not maintain user verification table.

#### Proof

A privileged insider of the system can obtain the stored credentials of registered user and perpetrate malicious attacks subsequently. However, during user registration phase of proposed protocol, *U*_*i*_ does not submit identity *ID*_*U*_ and password *PW*_*U*_ in plaintext form to the registration server *RS*. *U*_*i*_ submits only *A*_*U*_ = *h*(*PW*_*U*_ || *b*) and *PID*_*U*_ = *h*(*ID*_*U*_ || *b*) to *RS* instead of original credentials, where *b* is a randomly chosen number. Hence, an insider cannot obtain the original credentials of any user. In this way, the proposed protocol attains resistance to insider attacks.

In the proposed protocol, *AS* authenticates *U*_*i*_ by verifying the equivalence of the received message with the computed values i.e. *M*_1_ ≟ *h*(*PID*_*U*_ || *C*_*US*_ || *R*_*U*_ || *R*_*U*_′). Registration server *RS* is not involved in the authentication process, whereas password changing phase requires *RS*’s help. However, *RS* does not verify the legitimacy of *U*_*i*_ during this phase. Hence, *RS* does not require maintaining a database to store any kind of user’s credentials. An intruder cannot be able to determine any information about users, while the servers are not maintaining user verification tables. Thus, the servers are free from investing on their storage spaces.

#### Proposition 9

The proposed protocol is secure against denial-of-service attack.

An adversary may cause denial-of-service attack, when he/she intercepts a valid authentication request message and replays the same message to *AS*. We have taken following approaches to prove that the proposed protocol is secure against denial-of-service attack.

#### Proof a

Consider a scenario where an adversary replays previous captured authentication request {*AID*_*U*_, *M*_1_, *R*_*U*_} without any modifications. Upon receiving the request, *AS* computes *R*_*U*_*′ = x*_*S*_^.^ *R*_*U*_, *PID*_*U*_ = *AID*_*U*_ ⊕ *R*_*U*_′, and compares the extracted {*PID*_*U*_, *R*_*U*_} with the stored {*PID*_*U*_, *R*_*U*_}. When *AS* identify the received *R*_*U*_ is same as the stored *R*_*U*_ (i.e. *R*_*U*_ = = *R*_*U*_), then it can reject the request without even verifying *M*_1_ ≟ *h*(*PID*_*U*_ || *C*_*US*_ || *R*_*U*_ || *R*_*U*_′). This procedure can be completed with just one elliptic curve point multiplication operation and one X-OR operation.

#### Proof b

Assume that an adversary transmits fake requests such as {*AID*_*U*_^*#*^, *M*_1_^*#*^, *R*_*U*_^*#*^} for multiple. Upon receiving this message, *AS* computes *R*_*U*_′ *= x*_*S*_ · *R*_*U*_, *PID*_*U*_ = *AID*_*U*_ ⊕ *R*_*U*_′, *C*_*US*_ = *h*(*PID*_*U*_ || *K*_*S*_), and verifies *M*_1_ ≟ *h*(*PID*_*U*_ || *C*_*US*_ || *R*_*U*_ || *R*_*U*_′). It is obvious that the condition generates negative response due to unavailability of original *C*_*US*_ value with adversary. Therefore, *AS* believes it as a malicious attack and terminates the session, which requires two hash computations and one elliptic curve point multiplication operation.

#### Proposition 10

The proposed protocol provides forward secrecy.

#### Proof

Forward secrecy ensures that the session key remains safe, even though the long term private keys of communicating parties are compromised. The session key of the proposed protocol is computed as *SK* = *h*(*R*_*U*_′ || *C*_*US*_ || *SID*_*S*_ || *N*_1_) and the long term private key of the server *K*_*S*_ in *C*_*US*_ = *h*(*PID*_*U*_ || *K*_*S*_) is shielded with a hash function and is not possible to derive due to its one-way property. Although the long term key is compromised with an adversary; he/she still cannot construct a valid session key due to following reason. The parameter *R*_*U*_′ = *x*_*U*_ · *R*_*S*_ is dynamic due to its association with random generated number *x*_*U*_, which is not possible to extract due to the reason of ECDLP. Therefore, the proposed protocol provides perfect forward secrecy.

### Simulation for formal security verification using AVISPA tool

In this section, we simulate the proposed protocol using the widely accepted AVISPA for the formal security verification. For this purpose, we first provide a brief background of AVISPA tool and then the implementation details. We finally analyze the simulation results reported in this section. Note that AVISPA allows to verify whether a security protocol is safe or unsafe against replay and man-in-the-middle attacks. The main goal of the formal security verification simulation is to verify whether the proposed scheme is secure against replay and man-in-the-middle attacks.

#### Overview of AVISPA

AVISPA is a push-button tool for the automated validation of Internet security-sensitive protocols and applications. AVISPA is a widely-accepted and used tool to formally verify whether a cryptographic protocol is safe or unsafe against passive and active attacks including the replay and man-in-the-middle attacks [2], [52]. In AVIPSA, a security protocol is implemented using HLPSL (High Level Protocols Specification Language) [53], [54]. In HLPSL implementation, the basic roles are used for representing each participant role, and composition roles for representing scenarios of basic roles. The role system includes the number of sessions, the number of principals and the roles.

In HLPSL, an intruder (*i*) is modeled using the Dolev-Yao model [55] where the intruder can participate as a legitimate role. HLPSL is translated using HLPSL2IF translation to convert to the intermediate format (IF). IF is fed into one of the four backends: On-the-fly Model-Checker (OFMC), Constraint Logic based Attack Searcher (CL-AtSe), SAT-based Model-Checker (SATMC) and Tree Automata based on Automatic Approximations for the Analysis of Security Protocols (TA4SP). The detailed descriptions of these back-ends can be found in [54]. The output format (OF) is produced from IF by using one of these four back-ends. OF has the following sections [54]:

- SUMMARY: It indicates that whether the tested protocol is safe, unsafe, or inconclusive.
- DETAILS: It either explains under what condition the tested protocol is declared safe, or what conditions have been used for finding an attack, or finally why the analysis is inconclusive.
- PROTOCOL: It denotes the name of the protocol.
- GOAL: It indicates the goal of the analysis.
- BACKEND: It represents the name of the back-end used.
- At the end, after some comments and statistics, the trace of an attack (if any) is displayed in the standard Alice-Bob format.

There are several basic types supported in HLPSL, some of them are given below for better understanding of the implementation details in Section 7.2 [48]:

- *Agent*: It denotes the principal names. The intruder has always the special identifier *i*.
- *Public key*: It denotes agents’ public keys in a public-key cryptosystem. For example, given a public (respectively private) key *pk*, its inverse private (respectively public) key *pr* is obtained by *inv*(*pk*).
- *Symmetric key*: It means the keys for a symmetric-key cryptosystem.
- *Text*: It is often used as nonces. These values can be also used for messages.
- *Nat*: It denotes the natural numbers in non-message contexts.
- *Const*: It denotes the constants.
- *Hash*_*func*: It represents cryptographic hash functions.

In HLPSL, for concatenation the associative “.” operator is utilized. “*played*_*by X*” declaration means that the agent named in variable *X* plays in the role. A knowledge declaration (generally in the top-level *Environment* role) is used to specify the intruder’s initial knowledge. Immediate reaction transitions are of the form *X* = | > *Y*, which relates an event *X* and an action *Y*. By the goal *secrecy*_*of P*, a variable *P* is kept permanently secret. Thus, if *P* is ever obtained or derived by the intruder, a security violation will result.

#### Various roles implementation in HLPSL

We have three basic roles: *user* for a user *U*_*i*_, *registration server* for the registration server *RS* and *application server* for the application server *AS*. Besides these roles, the roles for the session, goal and environment in HLPSL are mandatory in the implementation. We have implemented the proposed protocol for user registration phase, login phase, and mutual authentication with key-agreement phase.

**Fig 5:**
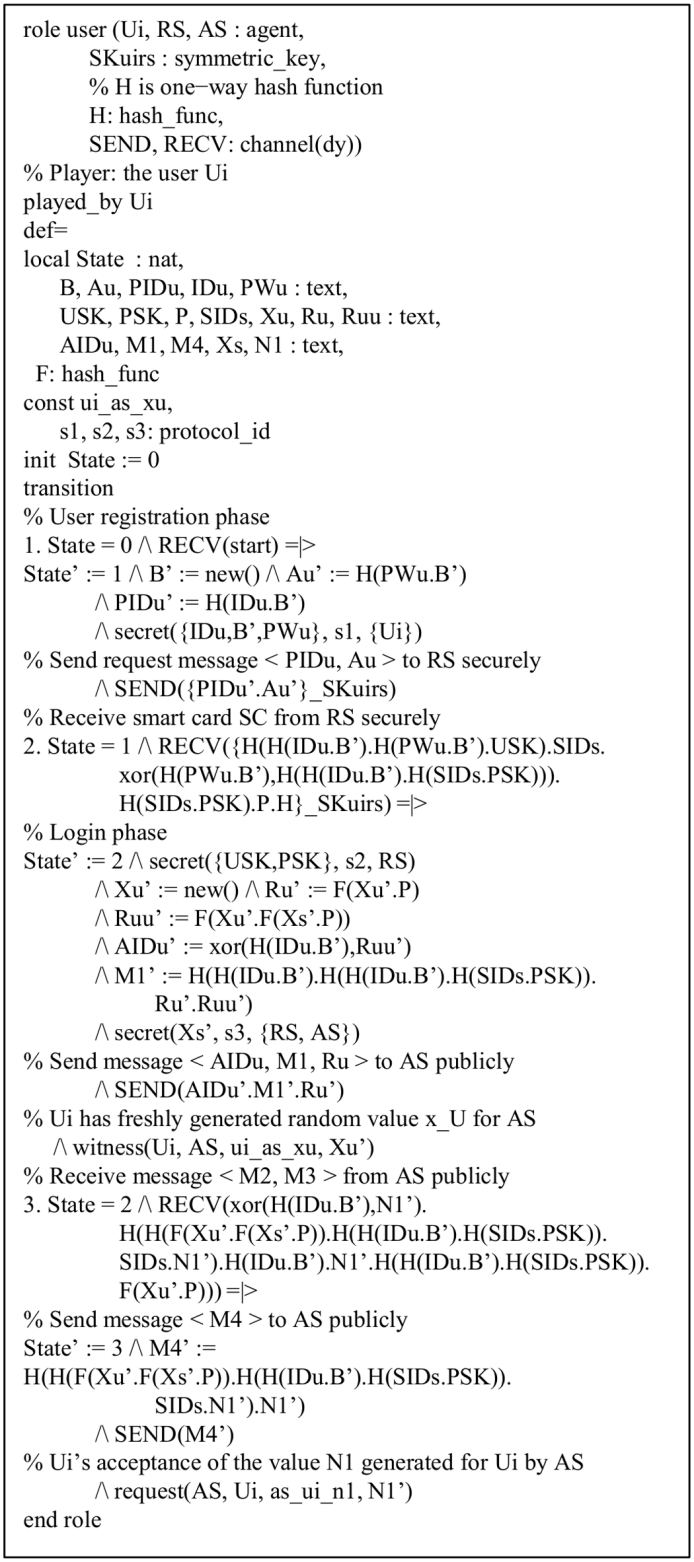
Role specification for user *U*_*i*_.

The role of the initiator, *U*_*i*_ is provided in Fig 5. *U*_*i*_ first receives the start signal, updates its state value from 0 to 1. The state value is maintained by the variable *State*. *U*_*i*_ sends the registration request message < *PID*_*U*_, *A*_*U*_ > securely to the *RS* during the user registration phase with the *SEND*() operation. *U*_*i*_ then receives a smart card *SC* containing the information {*B*_*U*_, *T*_*US*_, *P*, *h*()} securely from the *RS* by the *RECV*() operation, and updates its state from 1 to 2.

In the login phase, *U*_*i*_ sends the message < *AID*_*U*_, *M*_1_, *R*_*U*_ > to the *AS* via open channel. During the mutual authentication with key-agreement phase, *U*_*i*_ then receives the message < *M*_2_, *M*_3_ > from the *AS* and sends reply < *M*_4_ > to the *AS* via open channel.

Note that *channel* (*dy*) declares that the channel is for the Dolev-Yao threat model [55]. The intruder (*i*) can thus intercept, analyze, and/or modify messages transmitted over the open channel. witness(A, B, id, E) declaration denotes for a (weak) authentication property of *A* by *B* on *E*, declares that agent *A* is witness for the information *E*; this goal will be identified by the constant *id* in the goal section [48]. request(B, A, id, E) declaration represents a strong authentication property of *A* by *B* on *E*, declares that agent *B* requests a check of the value *E*; this goal will be identified by the constant id in the goal section [48]. For example, witness(U_i_, AS, u_i_ as x_u_, x_u_’) declares that *U*_*i*_ has freshly generated random number *x*_*U*_ for *AS*. By the declaration secret(ID_u_, B’, PW_u_, s1, U_i_), we mean that the information *ID*_*U*_, *b* and *PW*_*U*_ are kept secret to *U*_*i*_ only, which is identified by the protocol id s1.

**Fig 6:**
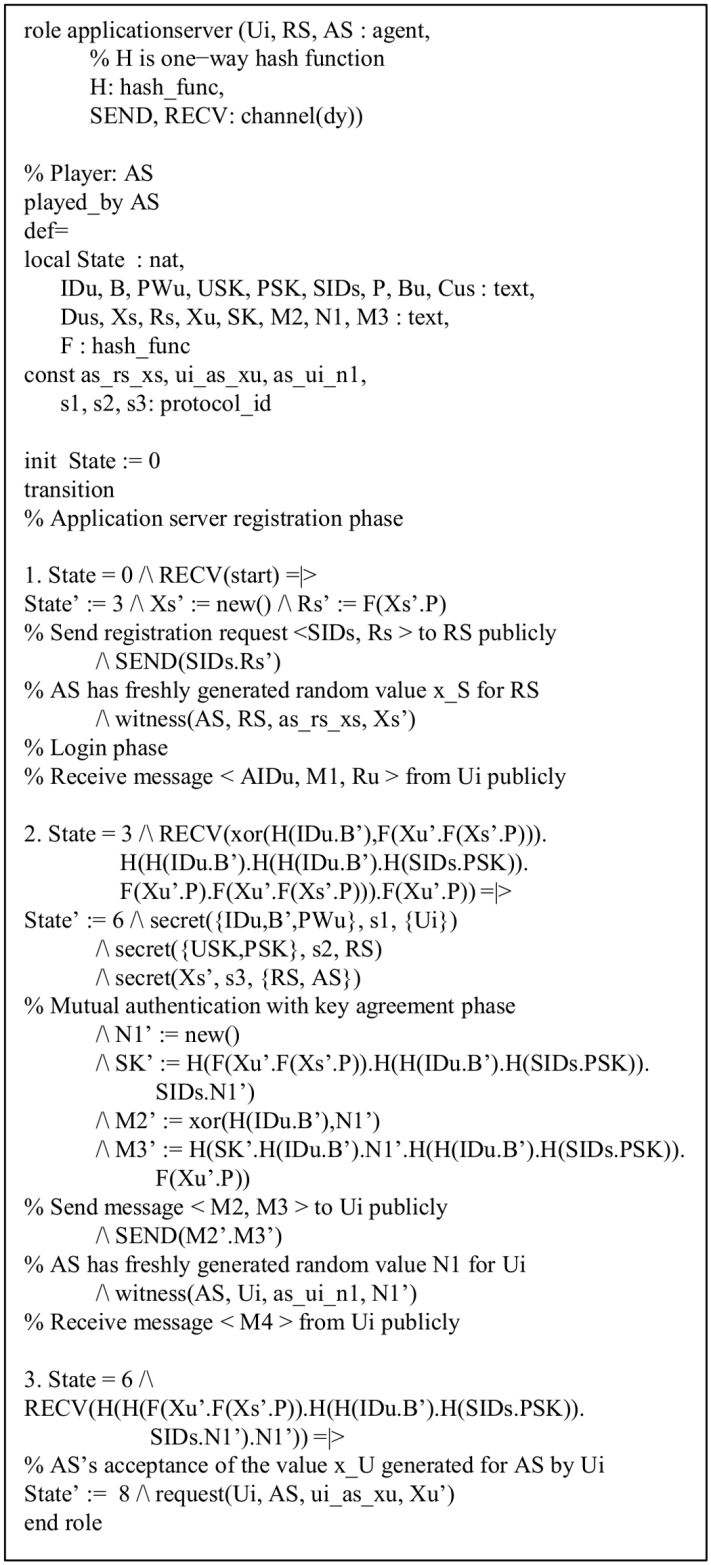
Role specification for application server *AS*.

**Fig 7:**
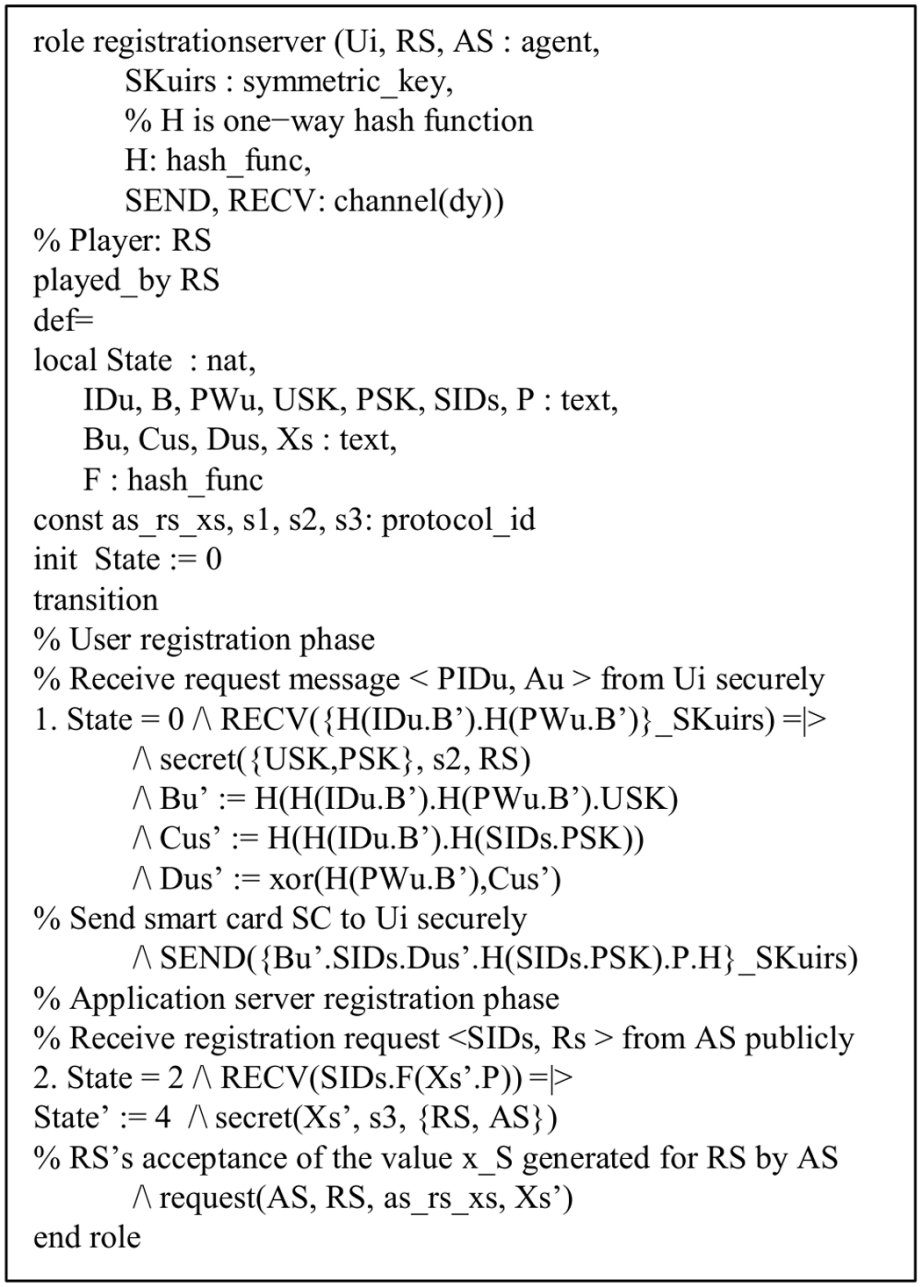
Role specification for registration server *RS*.

**Fig 8:**
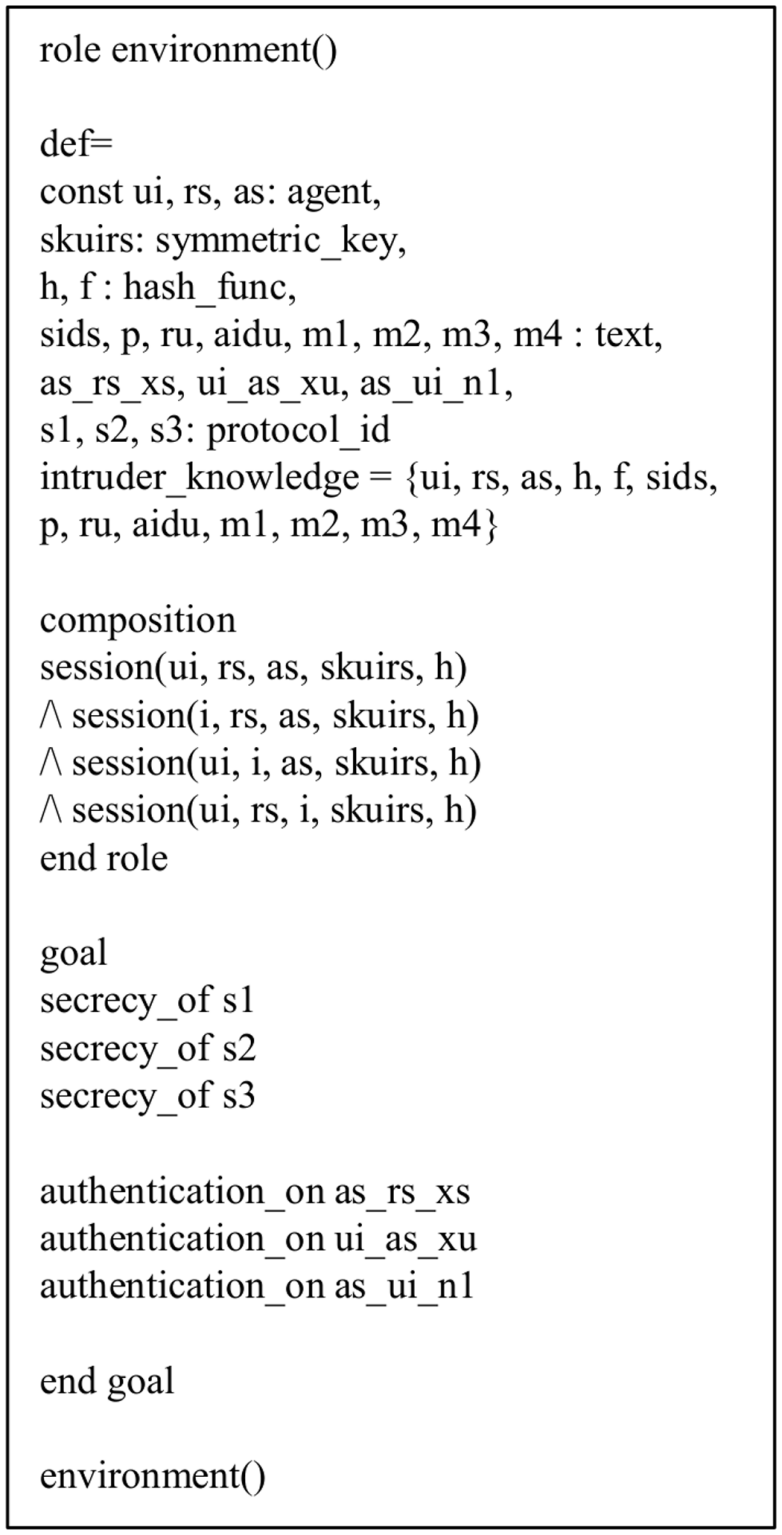
Role specification for the goal and environment.

**Fig 9:**
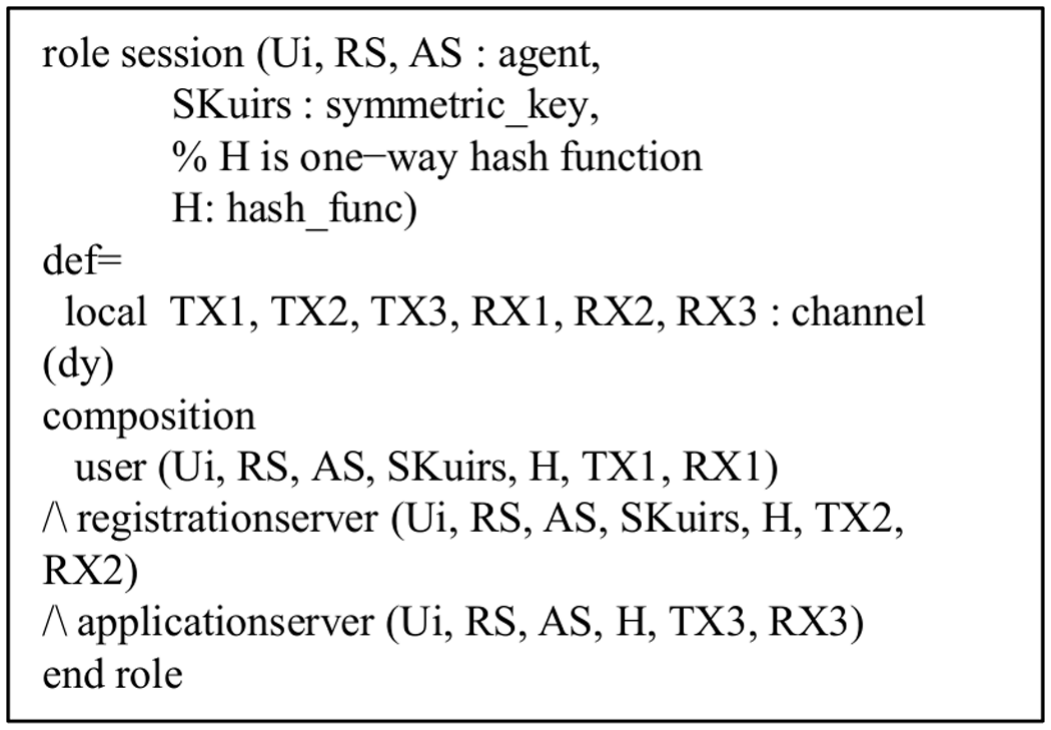
Role specification in HLPSL for the session.

In a similar way, the roles of the *AS* and *RS* of the proposed protocol are implemented and shown in Figs 6 and 7, respectively. The declaration, request(U_i_, AS, u_i_ as x_u_, x_u_’), signifies the *AS*’s acceptance of the value *x*_*U*_ generated for *AS* by *U*_*i*_. The roles for the goal and environment, and the session of the proposed protocol are also shown in Figs 8 and 9, respectively. In the session role, all the basic roles including *user*, *registrationserver* and *applicationserver* are the instances with concrete arguments. The top-level role (environment) is always specified in the HLPSL implementation. The intruder (*i*) participates in the execution of protocol as a concrete session as shown in Fig 8. In the proposed protocol, we have three secrecy goals and three authentication goals. For example, the secrecy goal: secrecy of s1 indicates that the information *ID*_*U*_, *b* and *PW*_*U*_ are kept secret to *U*_*i*_ only. The authentication goal: authentication_on ui_as_x denotes that the *U*_*i*_ has freshly generated random number *x* for the *AS*, where *x* is only known to *U*_*i*_. When the *AS* receives *x* from messages of *U*_*i*_, the *AS* checks a strong authentication for *U*_*i*_ based on *x*. Similarly, the other authentication goal authentication_on as_ui_n1 denotes that the *AS* generates a random number *N*_1_ for *U*_*i*_ and when *U*_*i*_ receives *N*_1_ from other messages from the *AS*, *U*_*i*_ checks a strong authentication for the *AS* based on *N*_1_.

#### Analysis of simulation results

The proposed protocol is simulated under the widely-accepted OFMC and CL-AtSebackends using the SPAN, the Security Protocol ANimator for AVISPA [56]. Both back-ends are chosen for an execution test and a bounded number of sessions model checking [53]. Since the AVISPA implementation of our scheme in HLPSL uses bit XOR operation, currently SATMC and TA4SP backends do not support this feature. Due to this reason, the simulation results under both SATMC and TA4SP backends becomes inconclusive, and we have ignored these results in this paper.

**Fig 10:**
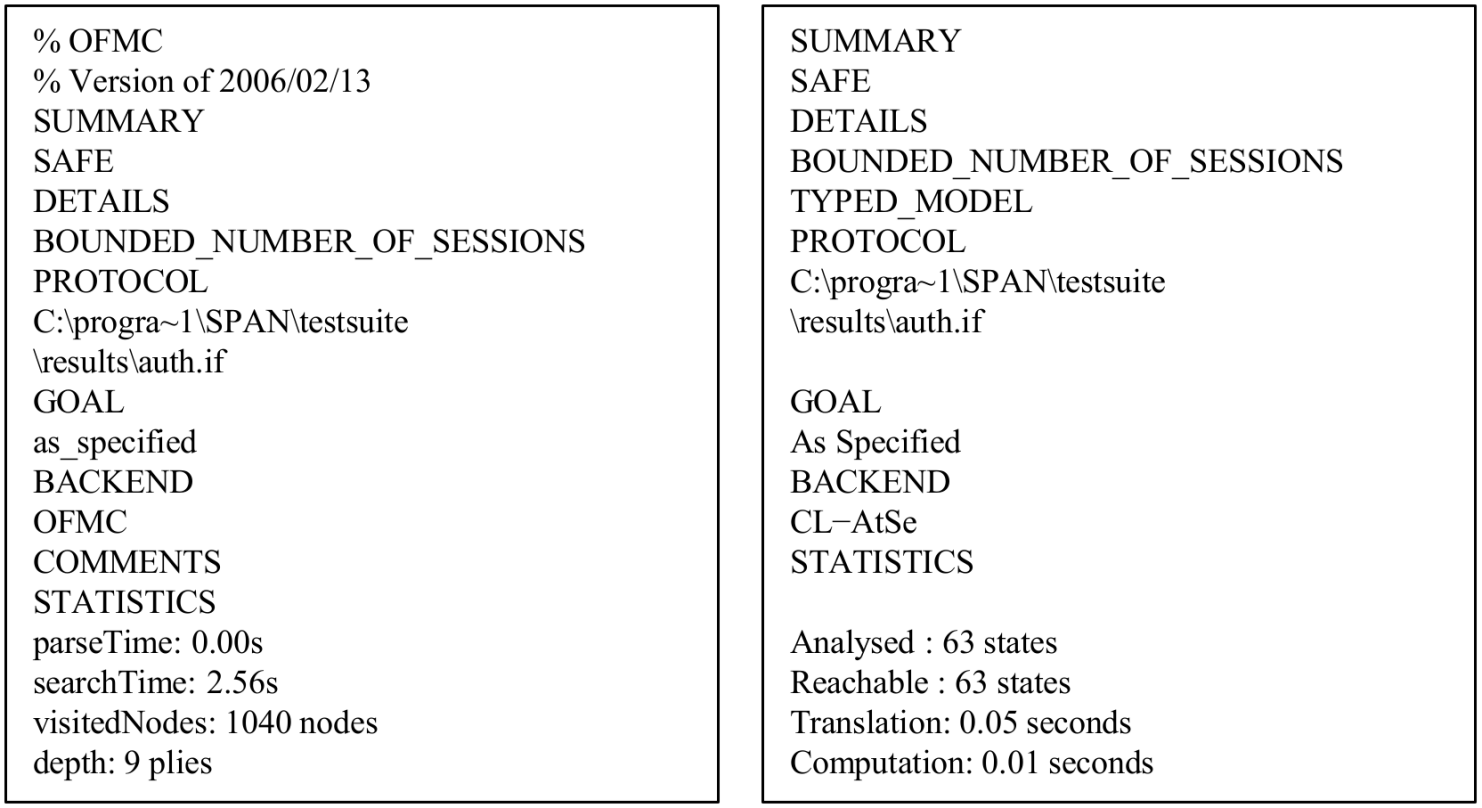
The result of the analysis using OFMC and CL-AtSe backends.

The following verifications are performed in the proposed protocol as in [57]:

- *Executability check on non-trivial HLPSL specifications*: Due to some modelling mistakes, the protocol model sometimes cannot execute to completion. It may be then possible that the backends cannot find an attack, if the protocol model cannot reach a state where that attack can happen. Therefore, an executability test is very essential in AVISPA [54]. The executability check of the proposed protocol tells that the proposed protocol description is well matched with the designed goals as specified in Figs 5–9.
- *Replay attack check*: For replay attack check, the OFMC and CL-AtSe back-ends verify if the legitimate agents can execute the specified protocol by performing a search of a passive intruder. Both backends provide the intruder the knowledge of some normal sessions between the legitimate agents. The test results reported in Fig 10 clearly indicate that the proposed protocol is secure against the replay attack.
- *Dolev*-*Yao model check*: For the Dolev-Yao model check, the OFMC and CL-AtSe backends also check if there is any man-in-the-middle attacks possible by the intruder. In OFMC backend, the depth for the search is nine and output of the results are shown in Fig 10. Also, the total number of nodes searched is 1040, which takes 2.56 seconds. On the other hand, in CL-AtSe backend, 63 states were analyzed and out of these states, all states were reachable. Further, CL-AtSe backend took 0.05 seconds for translation and 0.01 seconds for computation. It is clear from the simulation results that the proposed protocol fulfills the design criteria and is secure under the test of AVISPA using OFMC and CL-AtSe backends with the bounded number of sessions.

## Performance Analysis

This section demonstrates the performance analysis of the proposed protocol while considering various aspects such as security, computational cost and communication overhead. The performance analysis ensures that the proposed protocol is efficient and better in every aspect compared to Lu et al. [40] and other related protocols [2] [24] [25] [34] [39].

### Functionality comparison

In this subsection, the proposed protocol is evaluated in terms of security and compared with other similar authentication protocols for multi-server architecture. The comparison of security properties between Chuang et al. [25], Mishra et al. [2], Lin et al. [39], Chen et al. [24], Lu et al. [40] and the proposed protocol are portrayed in Table 3. As shown in Table 3, the proposed protocol can withstand various security attacks and accomplishes distinct features such as user anonymity, untraceability, no verification tables, and biometrics deployment.

**Table 3:** Comparison of security properties.

Security property	Chuang [25]	Mishra [2]	Lin [39]	Chen [24]	Lu [40]	Our
P1	No	No	No	Yes	No	Yes
P2	Yes	Yes	No	Yes	Yes	Yes
P3	Yes	Yes	Yes	Yes	Yes	Yes
P4	No	No	Yes	Yes	No	Yes
P5	No	No	Yes	Yes	Yes	Yes
P6	No	No	Yes	Yes	No	Yes
P7	No	No	Yes	Yes	No	Yes
P8	Yes	Yes	No	Yes	Yes	Yes
P9	No	Yes	No	Yes	Yes	Yes
P10	Yes	Yes	Yes	Yes	Yes	Yes
P11	Yes	Yes	No	Yes	Yes	Yes
P12	Yes	Yes	No	Yes	No	Yes
P13	Yes	Yes	Yes	Yes	No	Yes

P1: User anonymity and untraceability, P2: Perfect mutual authentication, P3: Prevent replay attack, P4: Prevent man-in-middle attack, P5: Prevent stolen smart card attack, P6: Prevent user impersonation attack, P7: Prevent server impersonation attack, P8: Prevent insider attack, P9: Prevent denial-of-service attack, P10: Prevent password guessing attack, P11: No user verification table, P12: Prevent clock synchronization problem, P13: Perfect forward secrecy

### Computational cost comparison

It is evident from Table 4 that the computational cost of the proposed protocol is relatively lesser compared to Lu et al.’s and other similar protocols while accomplishing the significant security level as shown in Table 3. The proposed protocol is built on simple elliptic curve cryptography operations, one-way hash functions, concatenation and exclusive-OR operations. The computations of an exclusive-OR function and concatenation operation are relatively negligible, whereas exponential operation, elliptic curve point multiplication, encryption and decryption operations consume quite more. Research has proven that there is always a trade-off between security and performance of a protocol. Usually, when the protocol becomes more secure, the computational cost becomes higher and vice versa. Contrarily, the proposed protocol succeeds to stabilize both the terms parallel. To evaluate the computational cost analysis, we give few notations for the involved actions in Chuang et al. [25], Mishra et al. [2], Lee et al. [34], Lin et al. [39], Chen et al. [24], Lu et al. [40] and the proposed protocol as shown below.

**Table 4:** Comparison of computational cost.

Phase	Chuang [25]	Mishra [2]	Lee [34]	Lin [39]	Chen [24]	Lu [40]	Our
Login	3*T*_*h*_	7*T*_*h*_	2*T*_*h*_+2*T*_*c*_	5*T*_*h*_+1*T*_*fun*_	3*T*_*h*_	5*T*_*h*_	5*T*_*h*_+2*T*_*mul*_
Authentication+key-agreement	16*T*_*h*_	17*T*_*h*_	12*T*_*h*_+4*T*_*c*_	10*T*_*h*_+4*T*_*mul*_+5*T*_*fun*_	16*T*_*h*_	13*T*_*h*_	8*T*_*h*_+1*T*_*mul*_
Total	19*T*_*h*_	24*T*_*h*_	14*T*_*h*_+6*T*_*c*_	15*T*_*h*_+4*T*_*mul*_+6*T*_*fun*_	19*T*_*h*_	18*T*_*h*_	13*T*_*h*_+3*T*_*mul*_

- *T*_*h*_: Time complexity of a one-way hash function
- *T*_*mul*_: Time complexity of a point multiplication operation on elliptic curve
- *T*_*fun*_: Time complexity of encryption or decryption function
- *T*_*c*_: Time for performing a chaotic map operation

### Communication overhead comparison

The communication overhead of the proposed protocol is compared with Chuang et al. [25], Mishra et al. [2], Lin et al. [39], Chen et al. [24], Lu et al. [40] and organized in Table 5. In order to evaluate the communication cost of the compared protocols, this paper considers SHA-1 hash function of 160 bits length, random number of 160 bits length, timestamp of 32 bits length, elliptic curve point of 160 bits length and 1024 bits modular prime for encryption and decryption function. As depicted in Table 4, the proposed protocol also uses 3 communication messages like the other similar protocols. In contrast, the proposed protocol requires only 960 bits for the 3 messages. Therefore, the proposed protocol consumes less bandwidth compared to Chuang et al. [25], Mishra et al. [2], Lin et al. [39], Chen et al. [24], Lu et al. [40] protocols.

**Table 5:** Comparison of communication overhead.

Feature	Chuang [25]	Mishra [2]	Lin [39]	Chen [24]	Lu [40]	Our
Number of messages	3	3	3	3	3	3
Number of bits	1280	1280	2528	1280	1216	960

## Conclusions

This paper reviewed the recently proposed Lu et al.’s protocol for multi-server architecture and demonstrated that their protocol contains several weaknesses. In addition, this paper proposed an enhanced biometric based authentication with key-agreement protocol for multi-server architecture based on elliptic curve cryptography using smartcards. The mutual authentication of the proposed protocol is proved using BAN logic and also achieved significant features such as user anonymity, no verification tables, biometric authentication, perfect forward secrecy, with less computational and communication cost. The formal security of the proposed protocol is simulated and verified using the AVISPA tool to show that the proposed protocol can withstand active and passive attacks. The proposed protocol is perfectly suitable for practical applications as it accomplishes simple elliptic curve cryptography operations, one-way hash functions, concatenation operations and exclusive-OR operations. The formal and informal security analyses and performance analysis sections of this paper showed that the proposed protocol performs better in every aspect compared to Lu et al.’s protocol and existing similar protocols.
